# Adaptive cellular evolution in the intestine of hyperdiverse cichlid fishes

**DOI:** 10.1038/s41586-026-10494-8

**Published:** 2026-05-13

**Authors:** Antoine Fages, Maëva Luxey, Fabrizia Ronco, Charlotte E. T. Huyghe, Sabrina Fischer, Gudrun Viktorin, P. Navaneeth Krishna Menon, Adrian Indermaur, Walter Salzburger, Patrick Tschopp

**Affiliations:** 1 Zoological Institute, Department of Environmental Sciences, University of Basel, Basel, Switzerland; 2 Present Address: MeLis, CNRS UMR 5284, INSERM U1314, Université Claude Bernard Lyon 1, Institut NeuroMyoGène, Lyon, France

**Keywords:** Molecular evolution, Evolutionary genetics

## Abstract

The ability of animals to efficiently track down and digest food is crucial for their survival, and the specialization to different diets is a major driver of diversification in this group^1–3^. Although the evolution of feeding structures has been studied extensively in this context^4–6^, the nature and extent of adaptations in the digestive tract remain poorly understood. Here, we examine dietary adaptations in the intestines of one of the largest adaptive radiations in vertebrates, the cichlid fishes of Lake Tanganyika. By generating comprehensive single-cell transcriptomic data for 24 Tanganyikan cichlid species with divergent feeding habits, and integrating this with eco-morphological and genomic information, we uncover that, at the cellular level, dietary adaptations primarily involve anterior enterocytes. In particular, we show that the relative abundances of anterior enterocytes as well as the gene expression profiles in this cell population evolved in response to rapid trophic specializations, and that these diet-related adaptations are driven by fast-evolving, cell-population-specific genes. Overall, our findings show that alterations in intestinal epithelium cell composition and in the cell-type-specific molecular makeup provided the substrate for trophic specializations, demonstrating that ecological adaptations target multiple layers of biological organization.

## Abstract (web summary)

Single-cell transcriptomics combined with morphological and ecological data show that the rapid evolutionary radiation of cichlid fishes in Lake Tanganyika was accompanied by dietary specialization across multiple layers of biological organization.

## Main

Dietary adaptations are paramount to the survival of animals, as illustrated by the remarkable diversity of feeding specializations, foraging behaviours, trophic morphologies and digestive physiologies in this group^7,8^. The nutritional process of animals involves two functionally and anatomically independent units, the feeding apparatus responsible for the uptake and mastication of food items, and the digestive tract responsible for enzymatic digestion and nutrient absorption^8^. The importance of adaptive modifications in the feeding apparatus for evolutionary diversification has been well documented across vertebrates and is particularly evident in cases of adaptive radiations, in which new species evolve rapidly from a common ancestor through adaptation to distinct ecological niches^9,10^. Famous examples include the varied beaks of Darwin’s finches^6^ and the diverse mouth and jaw morphologies of East African cichlid fishes^4,5^, which, in both cases, match the respective food sources and feeding habits^9^. Much less is known about the contribution of adaptations in the digestive tract in rapid ecological diversification. In this study, we capitalize on the extraordinary trophic diversity of the cichlid fish fauna of Lake Tanganyika^4,5^ to examine in detail and at the cellular level diet-related adaptations in the digestive tract. To investigate the cellular and molecular basis of dietary adaptations in the intestines of this rapidly diversifying group, we produced a phylogenetically comprehensive single-cell transcriptomic dataset, covering the digestive tracts of 24 ecologically distinct cichlid species from Lake Tanganyika. Through the integration of these data with extensive ecological and morphological information, we detect signatures of rapid dietary adaptations across multiple layers of biological organization and characterize the molecular underpinnings of cell type adaptations and tissue evolution in the context of this hyperdiverse vertebrate adaptive radiation.

## Cellular diversity in cichlid intestines

**Fig. 1:**
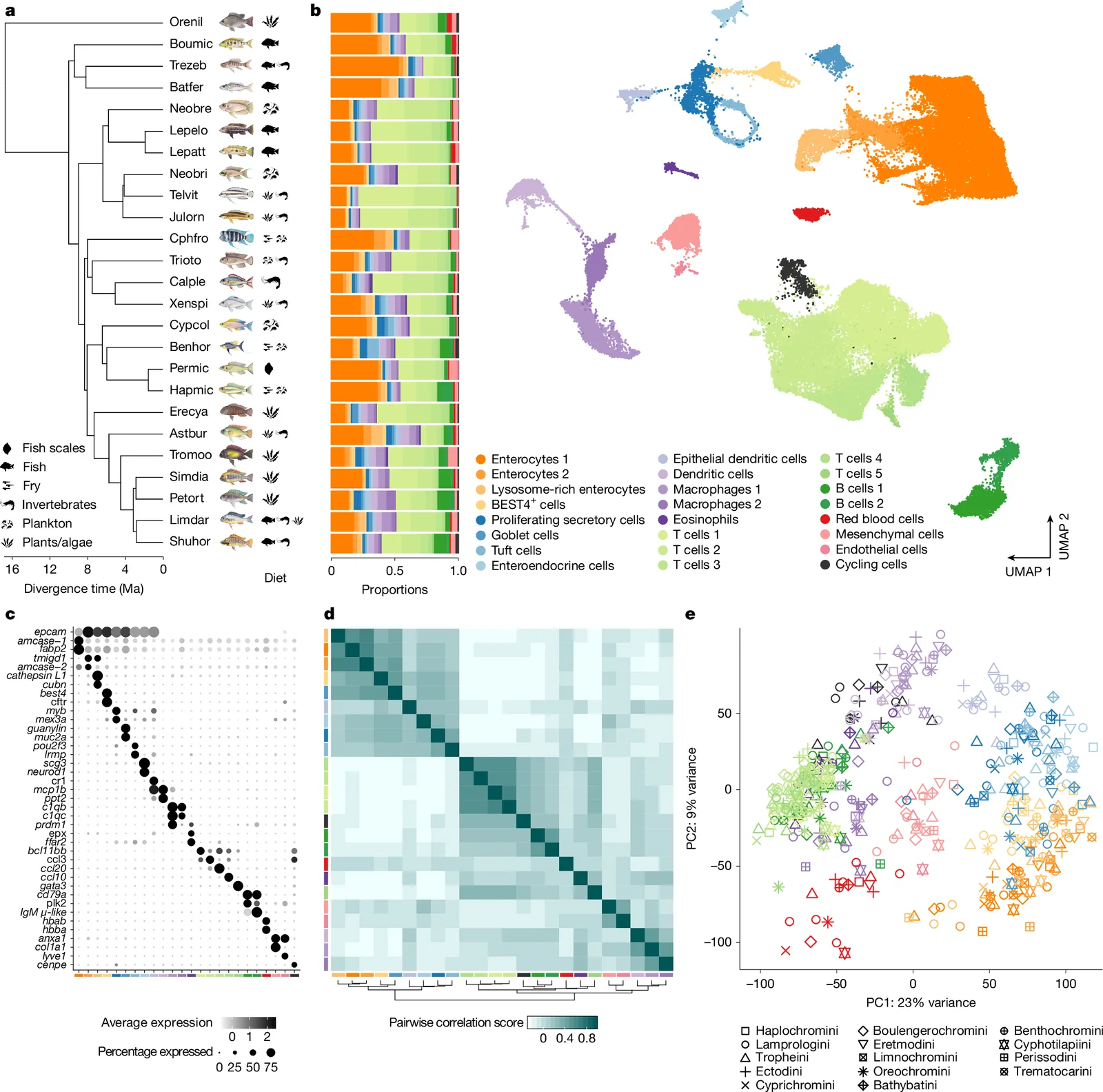
Characterization of the intestines of cichlid fishes at single-cell resolution. **a**, Time-calibrated genome-wide species tree of the 25 African cichlid species (24 from Lake Tanganyika, plus one outgroup species) included in this study, with trophic specializations indicated by symbols. Species names are abbreviated following the nomenclature detailed in ref. ^5^. **b**, Left, relative abundances of cell populations in the intestinal samples of each cichlid species; right, uniform manifold approximation and projection (UMAP) and cell-population-based clustering of 82,621 cells sampled from the intestines of all 25 cichlid species shown in **a**. **c**, Dot plot of selected marker gene expression, used to identify cell populations. **d**, Heatmap of pairwise gene expression correlations between cell populations. **e**, PCA of pseudobulk gene expression variation across all cell populations and species. In all panels, colours refer to cell populations, and shapes in **e** refer to the cichlid sub-clades (tribes) found in Lake Tanganyika. Fish illustrations in** a** reproduced with permission from Julie Johnson. Ma, million years ago.

**Extended Data Fig. 1:**
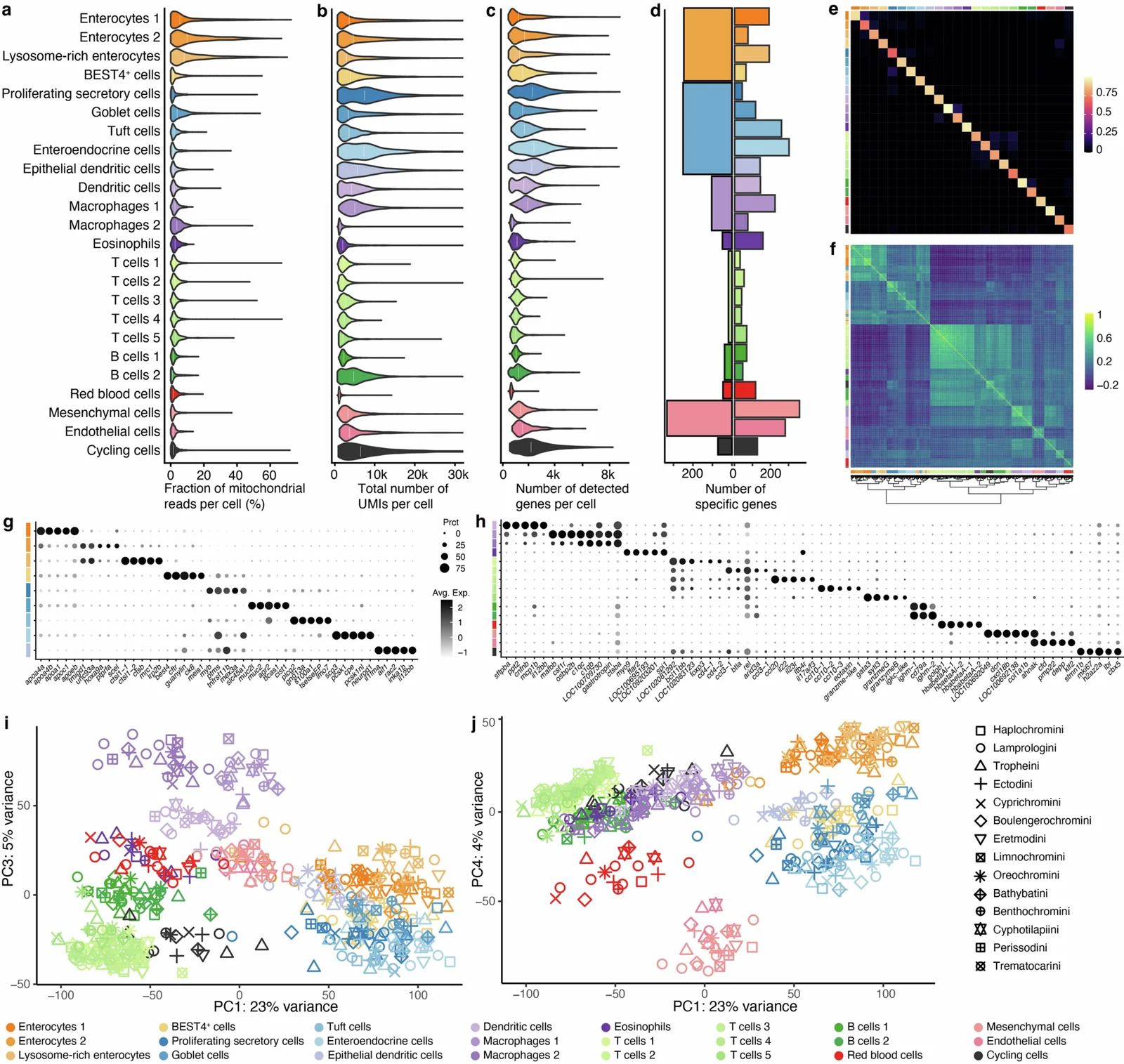
Characterization of intestinal cell populations. **a**, Fraction of reads mapping to mitochondrial genes in intestinal cell populations (means = 2 – 14%). **b**, Total number of unique molecular identifiers (UMIs) detected per cell in intestinal cell populations (means = 861 – 9157). **c**, Number of detected genes per cell in intestinal cell populations (means = 347 – 2148). In panels **a**, **b**, and **c**, vertical white lines mark the median values. **d**, Number of specific genes detected in intestinal cell populations (top) and cell type categories (down). **e**, Confusion heatmap of cross-validation results for the 24 distinct cell populations identified in the cichlid intestine. The gradient scale indicates the proportion of correct cross-validations, and ranges from 0 to 1. Row and column annotation colors represent the intestinal cell populations. **f**, Pairwise Spearman’s rank correlation matrix with hierarchical clustering of pseudobulks for each cell population and species in the dataset, ranging from 0 to 1. Row and column annotation colors represent the intestinal cell populations. **g**, Dot plot of expression of top five marker genes in epithelial cell populations. **h**, Dot plot of expression of top five marker genes in non-epithelial cell populations. **i**, PC1 and PC3 of PCA of pseudobulk gene expression variation across all cell populations and species. Colors refer to cell populations and shapes to the tribes to which the species belong. **j**, PC1 and PC4 of PCA of pseudobulk gene expression variation across all cell populations.

**Extended Data Fig. 2:**
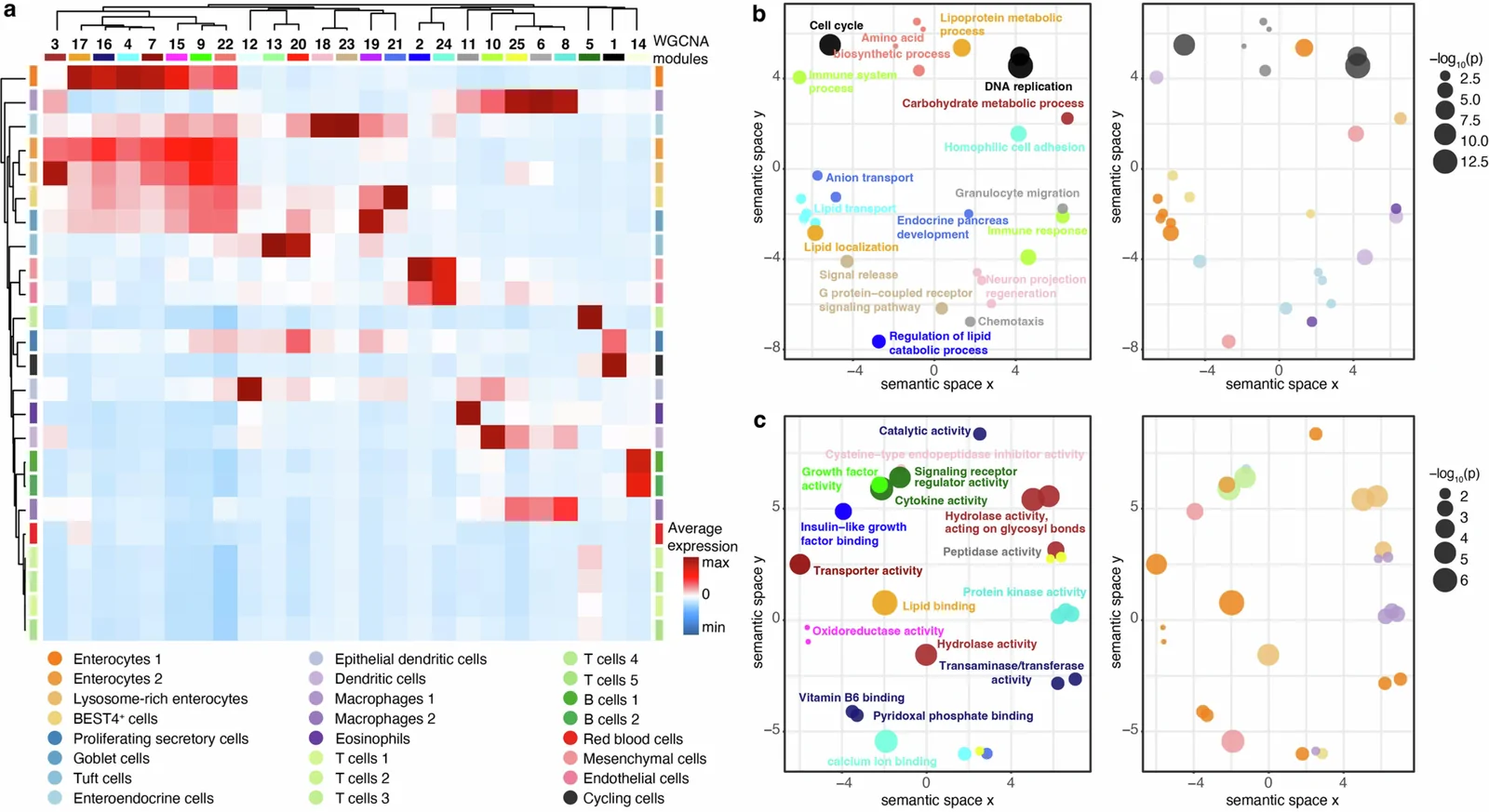
Modules of co-expressed genes in the intestines of cichlid fishes. **a**, Heatmap of average scWGCNA module eigengene expression across intestinal cell populations, scaled across scWGCNA modules, with associated hierarchical clusterings of cell populations and scWGCNA modules. Column annotation colors represent the scWGCNA modules, row annotation colors represent the intestinal cell populations. **b**, REVIGO scatterplot visualization of Biological Process gene ontology (GO) terms enriched in scWGCNA modules, colored by scWGCNA modules (left) or cell population of highest expression for each module (right). **c**, REVIGO scatterplot visualization of Molecular Function gene ontology (GO) terms enriched in scWGCNA modules, colored by scWGCNA modules (left) or cell population of highest expression for each module (right). In panels **b** and **c**, the sizes of the GO terms represent the P-values derived from GO-term enrichment analyses.

The cellular composition of the digestive tract is well documented in laboratory model organisms^11,12^, but remains unexplored in eminent ecological and evolutionary study systems such as the East African cichlid fishes. To scrutinize the tissue organization of the intestines of cichlid fishes, we generated single-cell RNA sequencing (scRNA-seq) data—pooled from multiple intestinal sections covering the foregut, midgut and hindgut—of 24 species endemic to Lake Tanganyika plus one non-endemic outgroup, the Nile tilapia (*Oreochromis niloticus*; Fig. 1a). The species were chosen to represent all 13 sub-clades (so-called tribes) of the radiation^5,13^, the main ecological niches that cichlids occupy in this lake, as well as the varied trophic specializations that they exhibit, ranging from pure herbivory or algivory to omnivory, invertivory, piscivory and scale-eating^5,14^. In total, in the 82,621 high-quality cells obtained from these experiments, we identified 24 cell populations with distinct transcriptomic signatures and molecular functions (Fig. 1b–d and Extended Data Figs. 1 and 2). The most abundant cell populations detected in the intestinal samples of cichlids were immune cells, consistent with the prominent role that the intestine has in the immune response of teleost fishes. Among cells of the digestive epithelium, defined here by the marker gene *epcam* (Fig. 1c), we detected all major absorptive and secretory cell types known across vertebrates, except for Paneth cells, which have not been observed in other teleost fishes either^15^. The most abundant epithelial cells were absorptive cells (Fig. 1b), which included a population of lysosome-rich enterocytes (LREs) specialized in protein uptake, previously described in zebrafish but absent in post-weaning mammals^16,17^.

**Extended Data Fig. 3:**
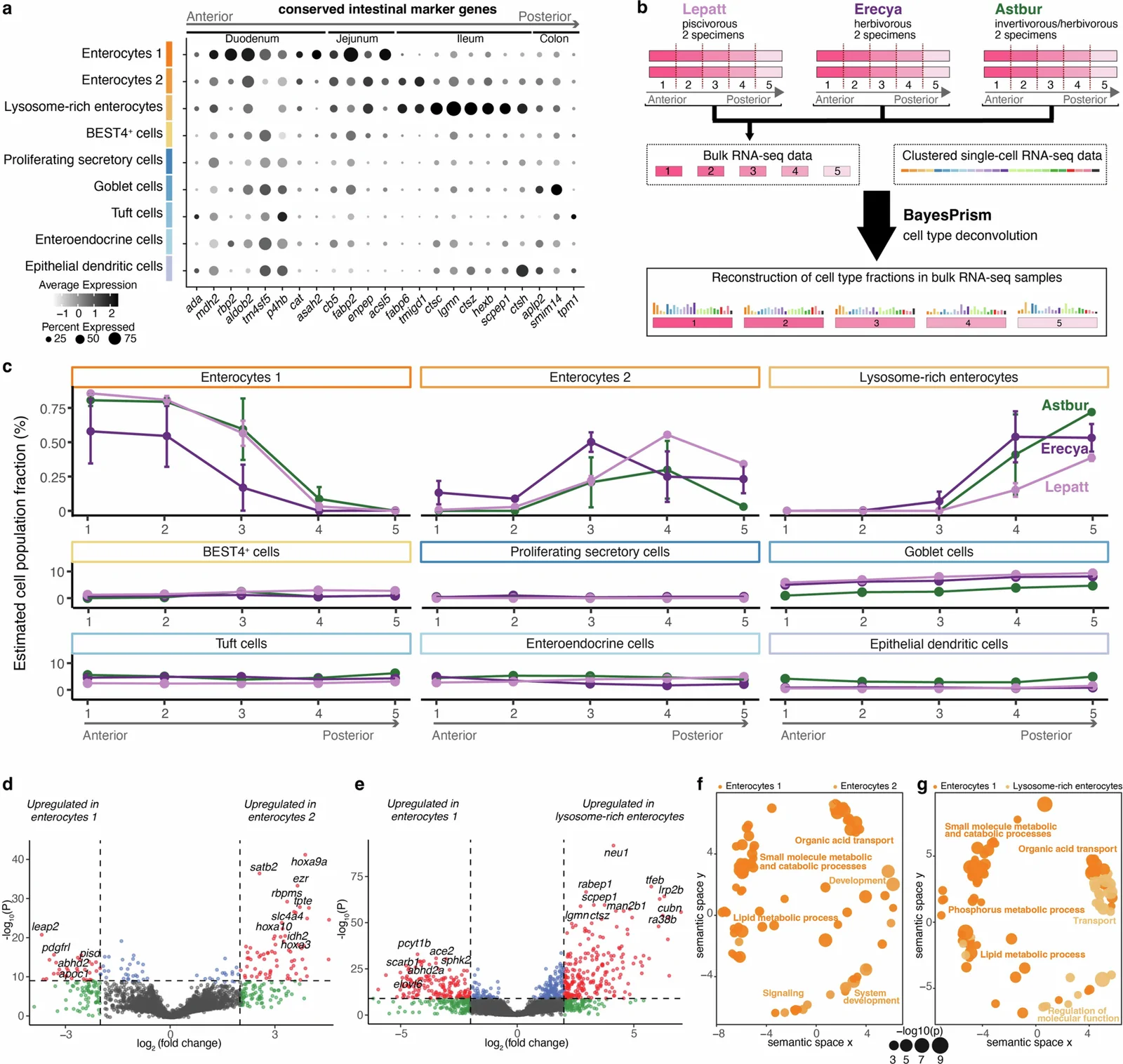
Anterior-posterior characterization of epithelial cell populations. **a**, Dot plot of expression of intestinal marker genes conserved in spatial patterns in mouse and zebrafish intestinal epithelia, as identified by Lickwar and colleagues^18^. **b**, Experimental design and workflow of cell type deconvolution of cichlid intestine bulk RNA-seq data informed by scRNA-seq data, using BayesPrism^19^. **c**, Estimated cell population fractions in the five intestinal parts of three African cichlid species, from duplicate biological samples for each species. The colors indicate the species’ tribe colors used in Fig. 3 and confidence intervals indicate standard error around mean values. **d** and **e**, Volcano plots of differential expression analysis between enterocytes 1 and enterocytes 2 (**d**), and enterocytes 1 and lysosome-rich enterocytes (**e**). Red dots indicate differentially expressed genes. **f** and **g**, REVIGO scatterplot visualizations of Biological Process GO terms enriched in differentially expressed genes between enterocytes 1 and enterocytes 2 (**f**), or in differentially expressed genes between enterocytes 1 and lysosome-rich enterocytes (**g**).

**Extended Data Fig. 4:**
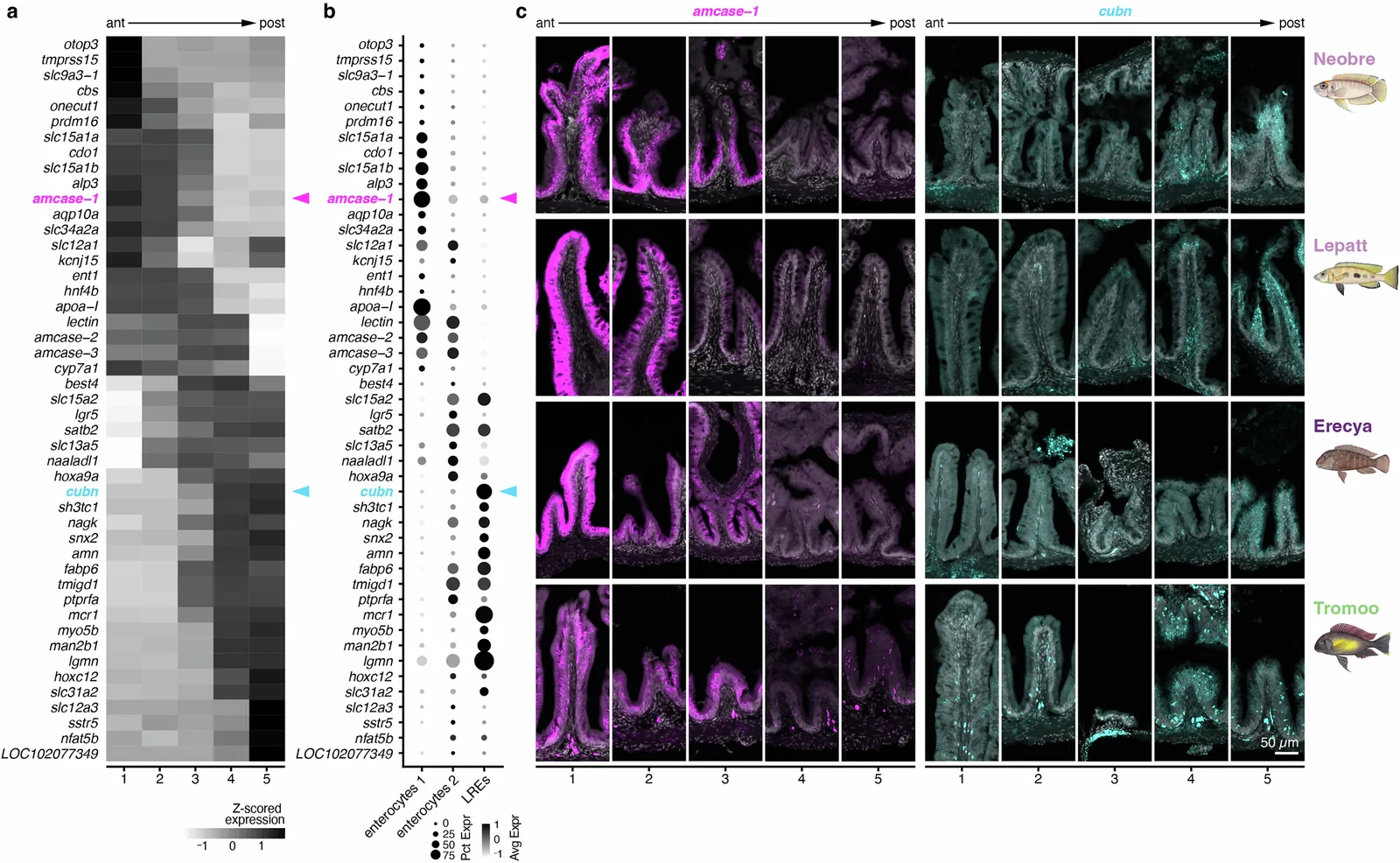
Experimental validation of anterior-posterior identity of enterocyte populations. **a**, Heatmap of Z-scored expression levels of the top differentially expressed genes across anterior-posterior regions in bulk RNA-seq data. The two genes used for fluorescent in situ hybridization in (**b**) are highlighted in color and by arrowheads. **b**, Dot plot of single-cell RNA-seq expression levels for the top differentially expressed genes along the anterior-posterior intestinal axis, separated by three spatially restricted enterocytes sub-populations. **c**, fluorescent in situ hybridization of *amcase-1* (left) and *cubn* (right) in five sections along the intestinal anterior-posterior axis of four cichlid species. Images are representative of two independent experiments, using at least duplicate biological samples for each species. 50 µm scale bar applies to all images. Fish illustrations in** c** reproduced with permission from Julie Johnson.

To approximate the anterior–posterior identity of our epithelial single-cell transcriptomes, we investigated the expression profiles of conserved region-specific intestinal markers^18^ (Extended Data Fig. 3a). Additionally, we sampled five sections along the intestines of three Tanganyikan cichlid species with divergent feeding ecologies and performed bulk RNA sequencing (RNA-seq). For each bulk RNA-seq sample, we estimated the relative abundance of all cell populations using a deconvolution approach^18,19^ (Extended Data Fig. 3b). We found that the anterior, intermediate and posterior intestinal regions of cichlids were enriched in enterocytes 1, enterocytes 2 and LREs, respectively (Extended Data Fig. 3c). Gene ontology (GO) enrichment analysis of differentially expressed genes across the enterocyte cell populations provided functional predictions consistent with this regional specialization (Extended Data Fig. 3d–g), which was experimentally confirmed through fluorescent in situ hybridization (ISH) experiments using marker genes for enterocytes 1 and LRE cells (Extended Data Fig. 4). Thus, although a clear-cut morphological compartmentalization of the intestine is not apparent in cichlid fishes^20^, we show that cichlids—just like other teleosts^16,18^—feature a cellular and molecular sub-functionalization along the anterior–posterior axis of their intestines.

To further characterize the identified cell populations in cichlids, we calculated pairwise gene expression correlations and found that cell populations cluster by broad cell type categories (Fig. 1d and Extended Data Fig. 1f). We next assessed interspecific gene expression divergence relative to cross-cell-population disparity by calculating pseudobulk values for each gene, cell population and species. A principal component analysis (PCA) based on the top 10,000 variable genes revealed that each cell type category and cell population can be well-characterized through the first four principal components (PCs), with PC1 separating epithelial cells from all other cell populations and PC2 to PC4 distinguishing absorptive from non-absorptive epithelial cells (Fig. 1e and Extended Data Fig. 1i,j). Importantly, the PCA also revealed that cross-species gene expression variation is primarily driven by differences between cell populations, and not by differences between species^21,22^. This shows that—at the within-tissue level, and after approximately 10 million years of divergence^4,5,13^—cell-population-specific gene expression differences dominate over species-level differences in Tanganyikan cichlids, thus permitting the identification of homologous cell populations and of potentially adaptive species-specific tissue compositions and gene expression patterns therein.

## Transcriptome evolution in the intestine

**Fig. 2:**
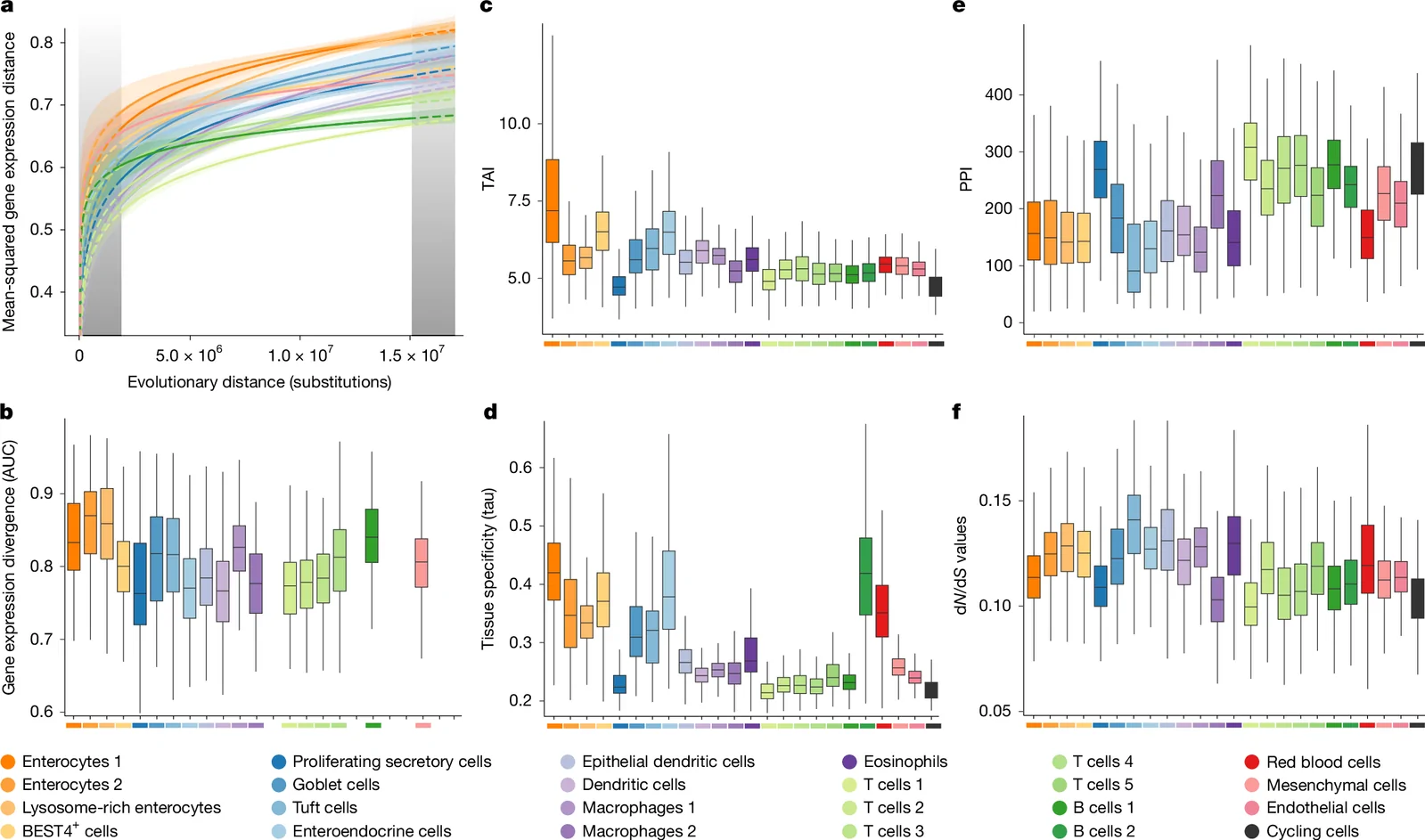
Cell-population-specific trajectories of transcriptome evolution in the cichlids’ intestines. **a**, Cell-population-resolved mean-squared gene expression distances as a function of evolutionary distances (in substitutions) calculated in EVEE-tools^24^, with 95% confidence intervals. **b**, Augur^28^ mean cross-validation AUC scores, ranging from 0.5 (random classifier) to 1 (perfect species identification of cells based on gene expression). **c**, TAI of 12,550 expressed genes. **d**, Tissue specificity (tau) scores of 25,687 expressed genes based on gene expression estimations from 13 cichlid tissues. **e**, Predicted number of PPIs of 13,977 expressed PCGs, based on the STRING database^34^. **f**, Quantification of signatures of selection in the coding sequences of PCGs, estimated by d*N*/d*S* of 4,172 expressed genes. Cell populations with a low number of cells or species represented were excluded from analyses shown in **a** and **b**, resulting in a total of 18 cell populations. In **b**–**f**, boxes show interquartile range, whiskers extend to values within 1.5 × interquartile range and horizontal lines mark the median values. AUC, area under the curve.

**Extended Data Fig. 5:**
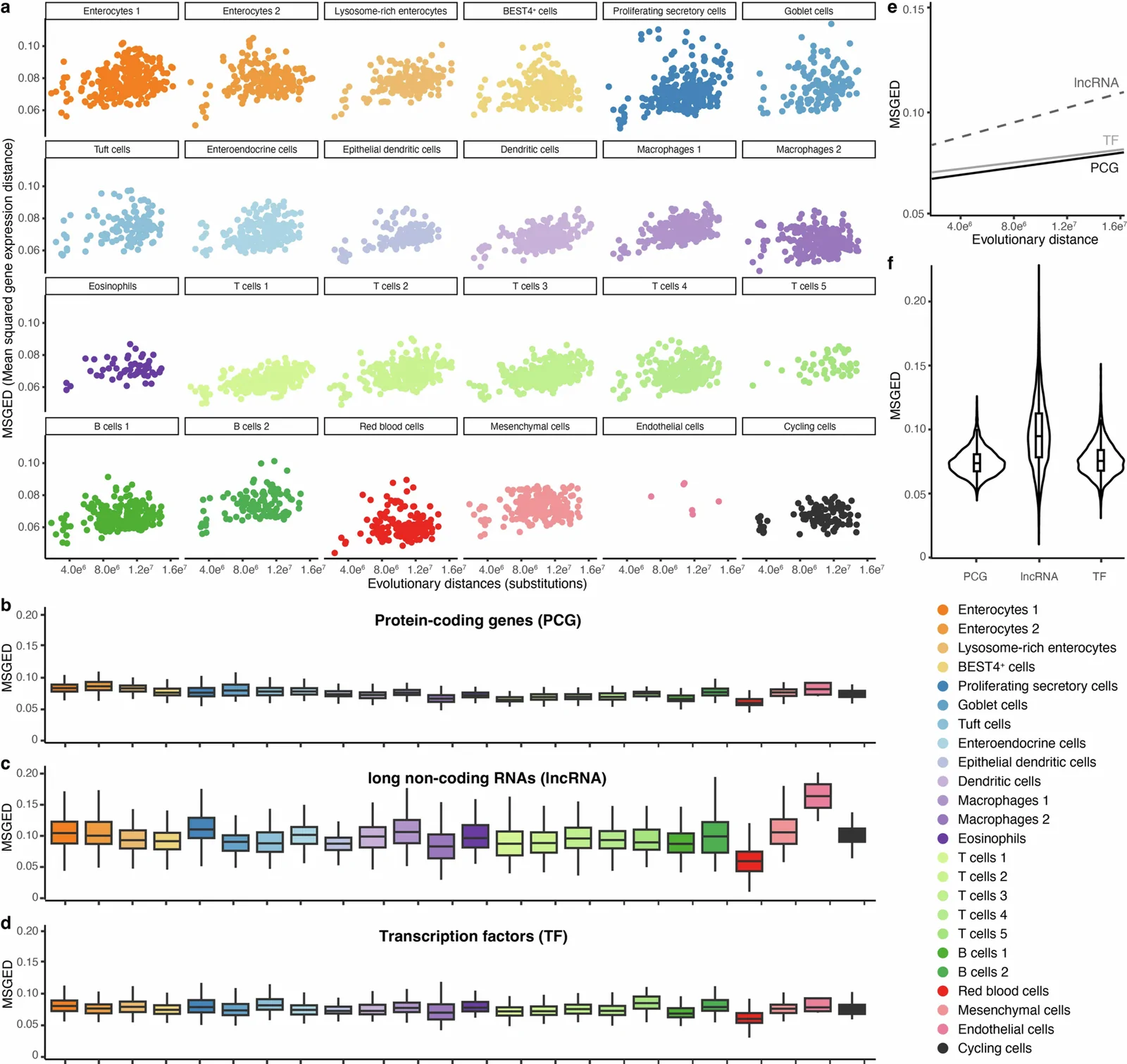
Cross-species gene expression divergence in intestinal cell populations. **a**, Mean-squared gene expression distances along evolutionary distances (measured in genomic substitutions) calculated across pairs of species in intestinal cell populations. **b**, Average mean-squared gene expression distances calculated between pairs of species and based only on protein-coding genes (PCGs) across all intestinal cell populations (n = 8,434 pairwise comparisons). **c**, Average mean-squared gene expression distances calculated between pairs of species and based only on long non-coding RNAs (lncRNAs) across all intestinal cell populations (n = 8,434 pairwise comparisons). **d**, Average mean-squared gene expression distances calculated between pairs of species and based only on transcription factors (TFs) across all intestinal cell populations (n = 8,434 pairwise comparisons). **e**, Mean-squared gene expression distances along evolutionary distances (measured in genomic substitutions) calculated across pairs of species, based on expression of PCGs (dark gray solid line), lncRNAs (gray dashed line) and TFs (light gray solid line). **f**, Average mean-squared gene expression distances calculated across pairs of species and based on expression of PCGs, lncRNAs and TFs. In panels **b**,**c**,**d**, and **f**, boxes display interquartile range, whiskers extend to values within 1.5 interquartile range, and horizontal lines mark the median values.

**Extended Data Fig. 6:**
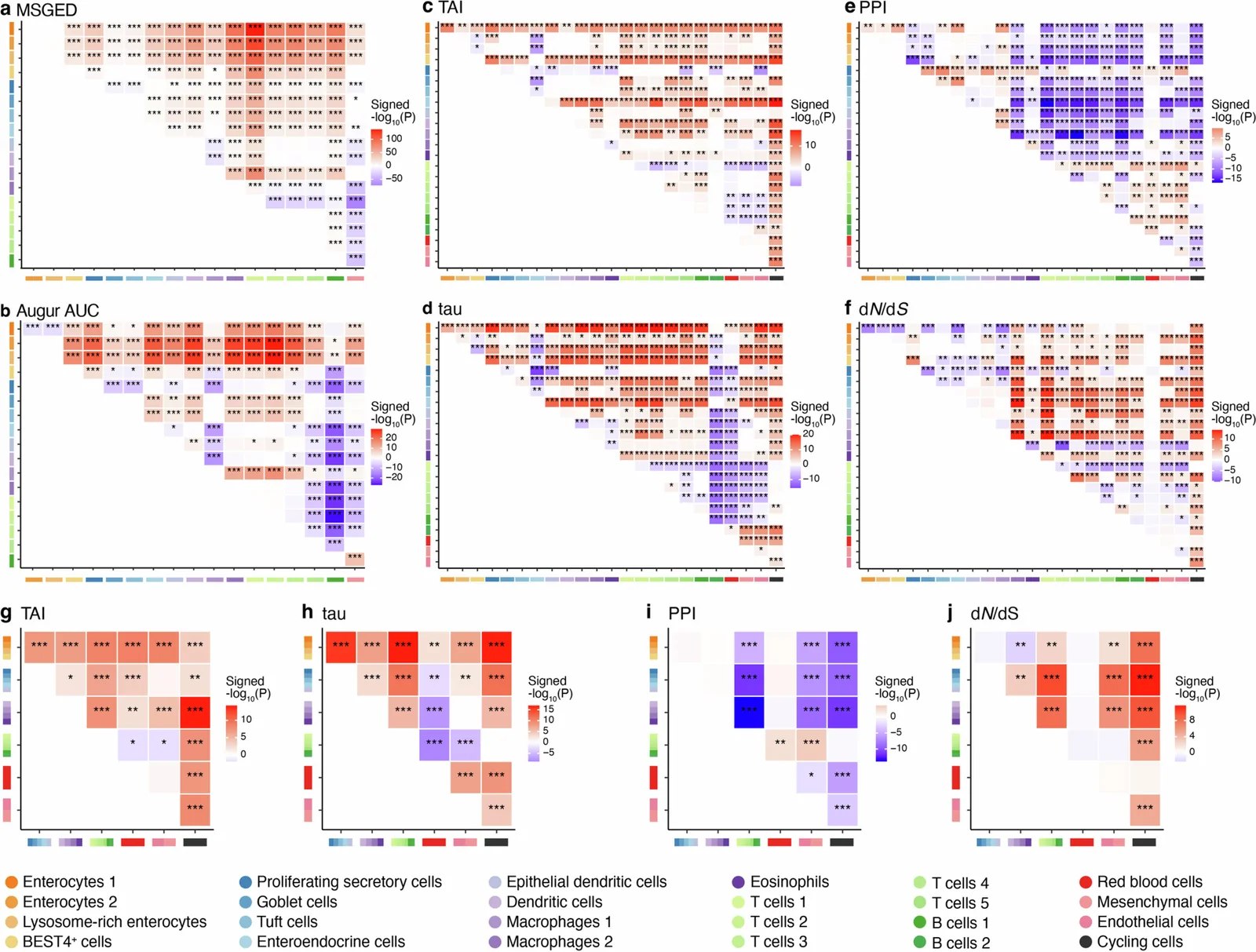
Statistical testing of differences in transcriptome characteristics across intestinal cell populations and cell type categories. **a**,**b**, Heatmap of P-values derived from paired two-sided Wilcoxon rank-sum tests across all pairs of cell populations. **c**–**f**, Heatmap of P-values derived from phylogenetic paired t-tests across all pairs of cell populations. **g**–**j**, Heatmap of P-values derived from phylogenetic paired t-tests across all pairs of cell type categories. Row and column colors refer to the cell populations or categories compared in a pairwise fashion. For each pairwise comparison and associated test, positive (negative) signed -log_10_(P) values indicate higher (lower) median value in the row cell population or cell type category than in the column one. *: P < 0.05, **: P < 0.01, ***: P < 0.001.

To characterize the evolutionary trajectories of the intestinal cell populations of cichlid fishes, we first investigated gene expression divergence for the 18 cell populations that contained at least 20 cells in two-thirds of the species. For each of these cell populations, we computed pseudobulk values and calculated mean-squared gene expression distances across all species pairs (Extended Data Fig. 5a). We found that gene expression divergence increases rapidly at short evolutionary distances between species, but saturates across longer distances, closely resembling an Ornstein–Uhlenbeck (OU) evolutionary process^23^ (Fig. 2a). This is in line with previous findings in mammals and fruit flies^24,25^, and corroborates the common view that gene expression in animals is constrained by stabilizing selection^21,26,27^. Interestingly, however, enterocyte cell populations exhibited greater gene expression differences across species than any other cell population, indicating increased divergence rates in these nutrient-absorbing cells (Fig. 2a and Extended Data Fig. 6a; two-sided Wilcoxon rank-sum tests, *P* < 0.001). By contrast, epithelial proliferating cells and non-epithelial cells consistently showed lower interspecific divergence in gene expression (Fig. 2a). This was confirmed by an independent machine-learning framework quantifying the extent of informative species-specific differentiation within each cell population and, therefore, how transcriptionally distinct cell populations are from species to species^28^ (Fig. 2b and Extended Data Fig. 6b). The differences in interspecific gene expression divergences between absorptive cells and the other cell populations were more marked in protein-coding genes (PCGs) than in both long non-coding RNAs and transcription factors (Extended Data Fig. 5b–d), whereas cross-species divergence was lower and increased slower with time in PCGs and transcription factors than in long non-coding RNAs (Extended Data Fig. 5e,f).

Next, we calculated the phylogenetic age index^29^ of each gene in each cell, to obtain a single transcriptome age index (TAI) for each cell population (Fig. 2c). Epithelial cells, and in particular enterocytes 1—the anterior-enriched absorptive epithelial cell type linked to lipid and small molecule metabolism (Extended Data Fig. 3f,g)—showed the highest TAI values (Extended Data Fig. 6c,g; phylogenetic paired *t*-tests, *P* < 0.01), indicating the expression of evolutionarily ‘young’ genes that may be subject to reduced evolutionary constraints^30^. By contrast, proliferating secretory cells and cycling cells had the lowest TAI, in line with the conservation of gene sets involved in cellular differentiation^31^ and cell cycle^32^. To estimate the extent of pleiotropic constraints in each cell population, we calculated the tissue specificity index for each gene, using available bulk RNA-seq data from 13 tissues (Fig. 2d). Tissue specificity was highest in absorptive epithelial cells and enteroendocrine cells, and low in proliferating secretory cells as well as in immune cells, mesenchymal cells and cycling cells (Extended Data Fig. 6d,h), consistent with the broad distribution of the latter cell populations across organs^33^. As another proxy for pleiotropic constraints, we inferred, for each PCG, the number of directly or indirectly inferred high-confidence protein–protein interactions (PPIs) from the STRING database^34^. We again found divergent patterns in intestinal cell populations, with most epithelial cells, macrophages and dendritic cells expressing PCGs with overall fewer PPIs than proliferating secretory cells, T cells, B cells and mesenchymal cells (Fig. 2e and Extended Data Fig. 6e,i; phylogenetic paired *t*-tests, *P* < 0.001). Tissue specificity and putative PPIs thus suggest that gene expression in epithelial cells, with the exception of proliferating secretory cells, has evolved under reduced pleiotropic constraints in cichlids.

Last, to examine selective pressures acting on the coding sequences of PCGs, we computed the ratios of non-synonymous over synonymous substitutions (d*N*/d*S* ratios) for PCGs across distant teleost lineages^35^ (Fig. 2f and Methods). We found that the mean d*N*/d*S* of absorptive and non-absorptive epithelial cells is higher than that of all other cell populations of the intestine (Extended Data Fig. 6f,j; phylogenetic paired *t*-tests, *P* < 0.01), indicating that the coding sequences of genes expressed in epithelial cells evolved under reduced selective constraints. Taken together, our findings suggest that although most intestinal cell populations have evolved under strong evolutionary constraints, absorptive epithelial cells—and in particular anterior-enriched enterocytes 1—show higher cross-species gene expression divergence, in line with relaxed phylogenetic and pleiotropic constraints.

## Adaptive cellular evolution in cichlids

**Fig. 3:**
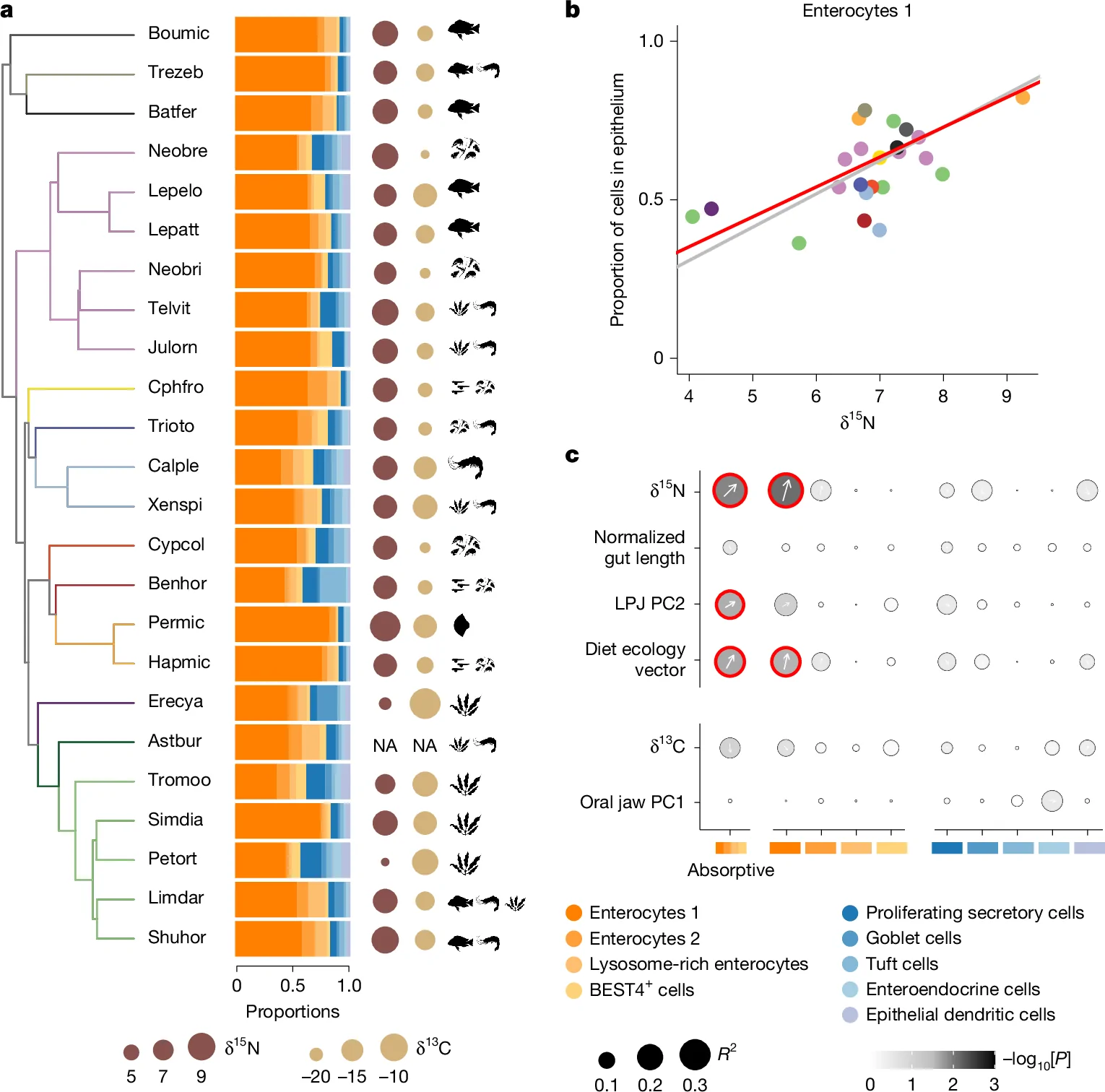
Associations between trophic specialization and epithelial cell population proportions. **a**, Left, time-calibrated genome-wide species tree of the 24 cichlid species from Lake Tanganyika included in this study. Branch colours refer to the cichlid tribes as detailed in ref. ^5^. Middle, relative abundance of absorptive (orange) and non-absorptive (blue) cell populations in the intestinal epithelium of each cichlid species. Right, stable nitrogen isotope (δ^15^N value) and stable carbon isotope (δ^13^C value) signatures estimated for each species^5^, with trophic specializations indicated by symbols. **b**, lm (grey) and pGLS (red) analyses showing a strong correlation between δ^15^N values and the relative abundance of enterocytes 1 (lm: *P* = 3 × 10^−3^, *R*^2^ = 0.35; pGLS: *P* = 4 × 10^−3^, *R*^2^ = 0.34, *λ* = 0.44. The colours of the points refer to the different cichlid tribes as in **a**. **c**, Summary dot plot of phylogenetic regression (pGLS) results between six ecological predictors, indicative of diet (top) or foraging ecology (bottom), and the centred log-ratios of relative abundance of epithelial cell populations. Statistically significant correlations after adjusting for false discovery rate (*P* < 0.05) are indicated with a red outline, and the arrows indicate the direction of the correlation (arrows pointing upwards and downwards indicate the slopes of positive and negative correlations, respectively). NA, stable isotope data not available.

**Extended Data Fig. 7:**
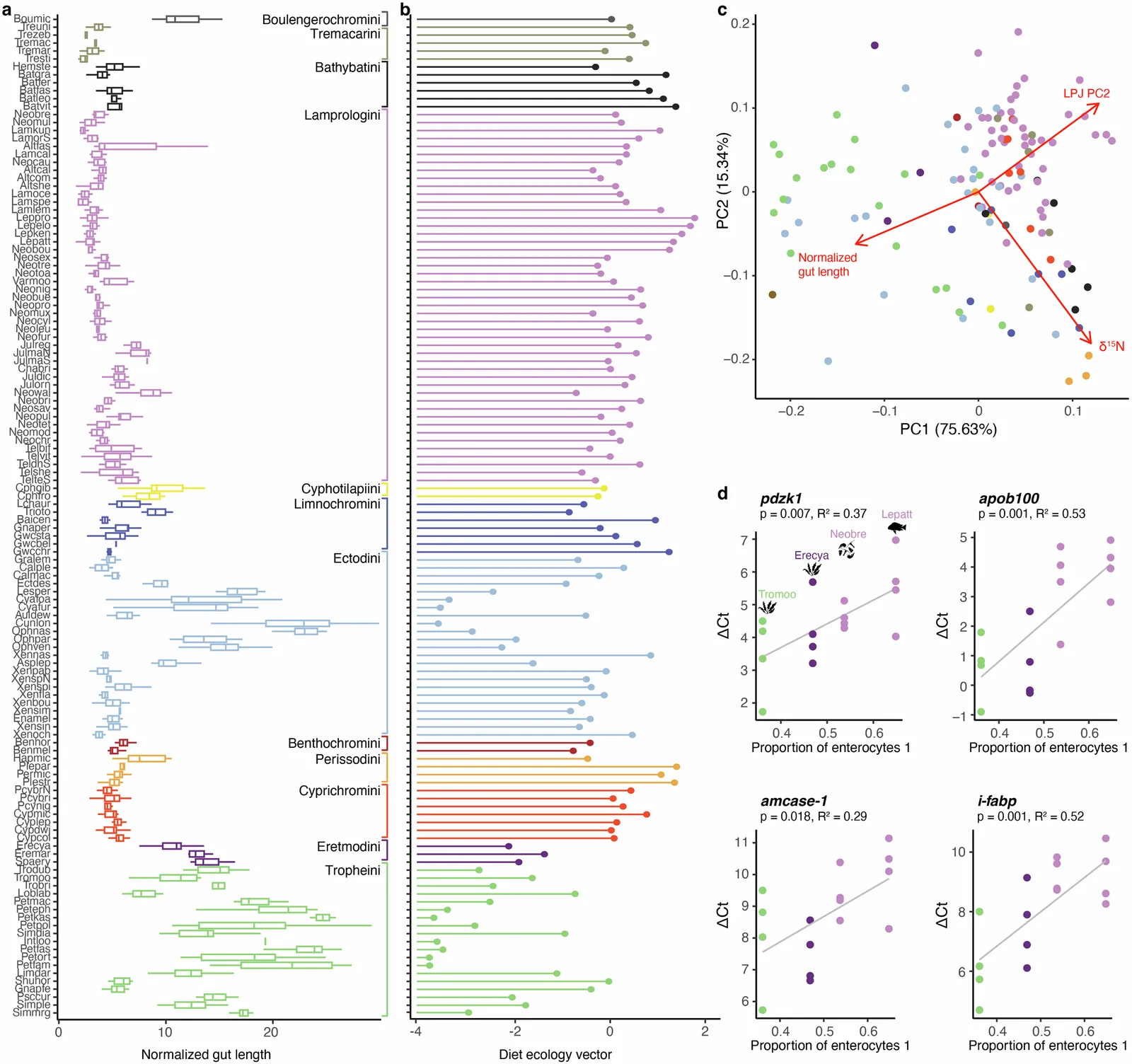
Ecological predictors and their associations with intestinal epithelium gene expression signatures. **a**, Normalized gut length, calculated as the ratio of total gut length to the cubic root of the weight measured in wild-caught specimens of 126 Tanganyikan cichlid species. Boxes display interquartile range, whiskers extend to values within 1.5 interquartile range, and horizontal lines mark the median values. **b**, PCA based on three dietary predictors (δ^15^N, normalized gut length and PC2 of LPJ morphology) measured across 126 Tanganyikan cichlid species. **c**, PC1 of the PCA shown in panel **b**, used as diet ecology predictor in ecological association analyses. **d**, Quantification of expression of marker genes of absorptive cells (top, *pdzk1* and *apob100*) and of marker genes of enterocytes 1 (bottom, *amcase-1* and *i-fabp*) through reverse transcription quantitative PCR (RT-qPCR), using the pan-epithelial gene *epcam* as reference, in four cichlid species with distinct dietary ecologies and relative abundances of enterocytes 1, as predicted by scRNA-seq quantifications (‘Proportion of enterocytes 1’). Linear model (lm, grey lines) regressions indicate a significant correlation between ΔCt and proportion of enterocytes 1 (P < 0.02, R^2^ > 0.29).

Variation in the abundance of homologous cell types across species has been documented in various tissues^36,37^. However, it remains elusive in most cases whether or not this type of variation is adaptive and can contribute to tissue specialization and, consequently, to organismal diversification. To test for phenotype–environment correlations at the cellular level, we contrasted relative cell type abundances in the cichlids’ intestinal epithelia with previously established proxies for foraging ecology and dietary niches^5,38^ (Fig. 3a). As ecological variables, we used stable carbon isotope signatures (δ^13^C values) as a proxy for the benthic–pelagic habitat axis, stable nitrogen isotope signatures (δ^15^N values) as a proxy for the trophic level (Fig. 3a) and eco-morphological covariates—namely, normalized gut length, oral jaw and lower pharyngeal jaw (LPJ) morphologies, as well as a vector summarizing the shared variation in dietary variables (Fig. 3b,c, Extended Data Fig. 7a–c and Methods). We found positive correlations between the relative abundance of absorptive epithelial cells and δ^15^N values across species (Fig. 3c; linear model (lm) and phylogenetic generalized least squares (pGLS): *R*^2^ = 0.37, adjusted *P* = 0.01, *λ* = 0). This trend is largely driven by an overrepresentation of enterocytes 1 in the intestines of cichlid species at higher trophic levels, that is, piscivores including scale eaters, and a relative underrepresentation of this cell population in species feeding on algae and plants (Fig. 3b,c; enterocytes 1, lm: *R*^2^ = 0.34, *P* = 0.02; pGLS: *R*^2^ = 0.40, *P* = 0.008, *λ* = 1). An orthogonal approach, namely, reverse transcription quantitative PCR (RT–qPCR) experiments, confirmed these cross-species differences in relative cell type abundances (Extended Data Fig. 7d; lm: *R*^2^ > 0.29, *P* < 0.02). Similarly, our bulk RNA-seq deconvolution approach revealed higher abundance of enterocytes 1 in the two most anterior gut sections of the piscivore compared with the two other species that feed on plants, diatoms and small arthropods (Extended Data Fig. 3c). Thus, our results uncover a phenotype–environment correlation between epithelial cell type composition and trophic niches in the adaptive radiation of cichlid fishes in Lake Tanganyika.

**Extended Data Fig. 8:**
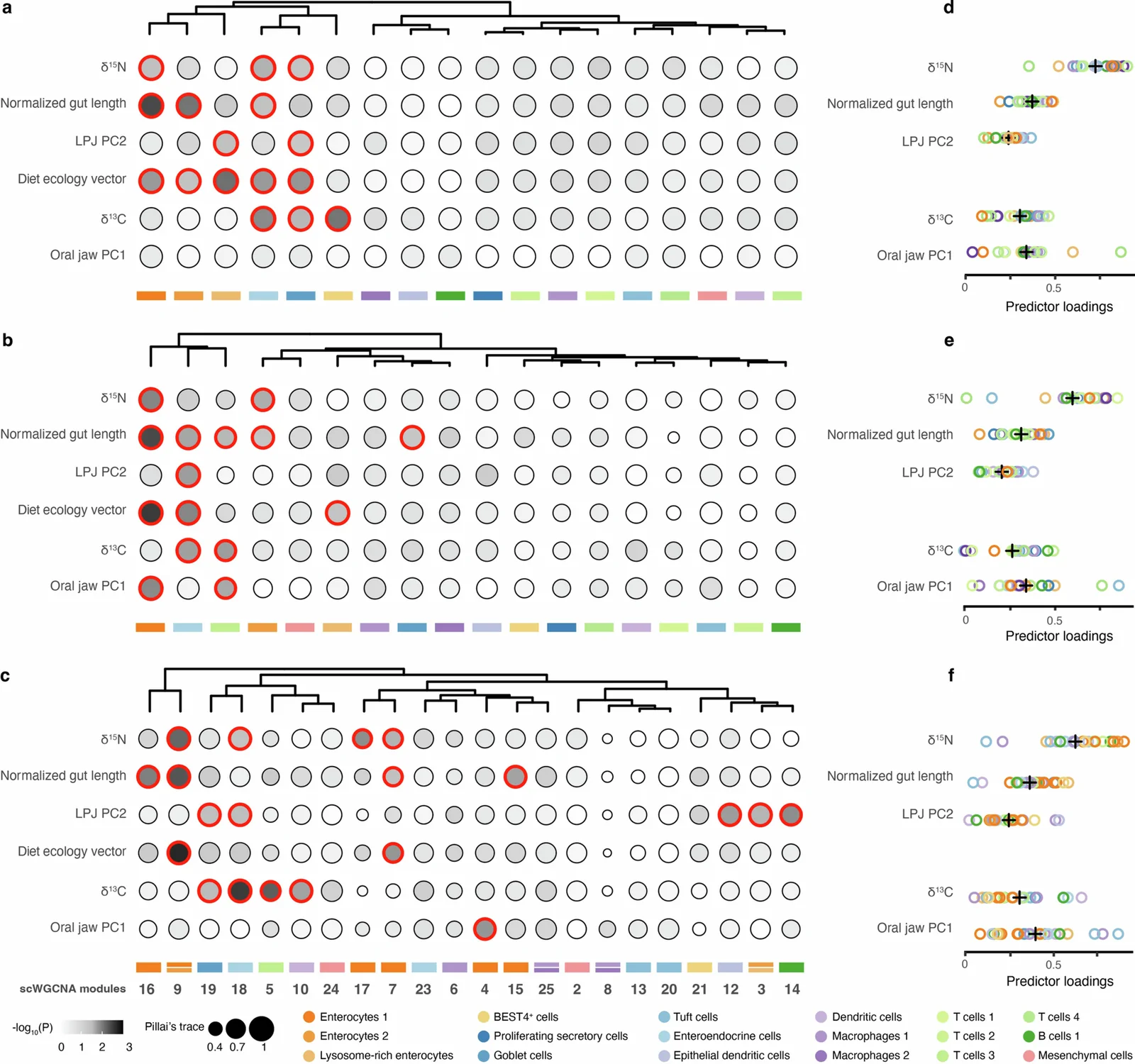
Correlations between gene expression signatures in intestinal cell populations and ecology. **a**, **b**, and **c**, Summary dot plots and hierarchical clustering of multivariate phylogenetic regressions (type II phylogenetic MANOVA, computed with mvMORPH) between six ecological predictors and gene expression in cell populations based on genes expressed in at least 20% of the cells within each cell population (**a**), cell population-specific genes (**b**), and genes in scWGCNA co-expression modules (**c**). Size of circles indicate Pillai’s trace values^101^, grayscale gradient indicates -log_10_(P), with statistically significant correlations (P < 0.05) indicated with a red outline, and hierarchical clustering based on -log_10_(P). **d**, **e**, and **f**, Ecological predictor loadings of a phylogenetic two-block partial least-squares (p2B-PLS) analysis of ecological predictors and cell population gene expression values. Black crosses indicate the mean predictor loadings across all cell populations. **d** is based on genes expressed in at least 20% of the cells within each cell population, **e** on cell population-specific genes, and **f** on genes in scWGCNA co-expression modules.

To further characterize the cellular and molecular responses to trophic niche adaptations, we examined the association between different gene expression signatures and ecological predictors. For each cell population, we performed multivariate phylogenetic regressions^39^ (see Methods for details) in three sets of genes, representing broadly expressed genes detected in at least 20% of cells (Extended Data Fig. 8a), cell-population-specific genes (Extended Data Fig. 8b) and genes co-expressed in single-cell weighted gene co-expression network analysis (scWGCNA) modules^40^ (Extended Data Fig. 8c), respectively. In the ‘global’ dataset of broadly expressed genes, ecological associations largely reflected overall gene expression affinities across cell populations, with most of the epithelial cell populations showing similar correlations with ecological predictors (Extended Data Fig. 8a). Analyses of more restricted gene sets showed stronger ecological signals within specific subsets of epithelial cells (Extended Data Fig. 8b,c). In particular, enteroendocrine cells showed strong signals of associations with both foraging ecology and diet, whereas enterocytes 1 showed strong and significant associations of gene expression signatures with several dietary predictors (δ^15^N values, normalized gut length and diet ecology vector) in all three sets of genes (Extended Data Fig. 8). This dietary signature was confirmed by a phylogenetic two-block partial least-squares analysis (p2B-PLS), which showed that δ^15^N values and normalized gut length were the primary ecological predictors of gene expression variation in enterocytes 1 (Extended Data Fig. 8d–f; two-sided Wilcoxon rank-sum test across the three gene sets, *P* < 4 × 10^−4^). By contrast, other enterocyte populations showed weaker and mostly ‘global’ dataset-specific associations with ecological factors, indicating a less pronounced cell-population-specific involvement in adaptations to different dietary niches. This again emphasized the pivotal role of enterocytes 1 in dietary adaptations in the intestinal epithelium of Tanganyikan cichlids.

## Molecular basis of dietary adaptations

**Fig. 4:**
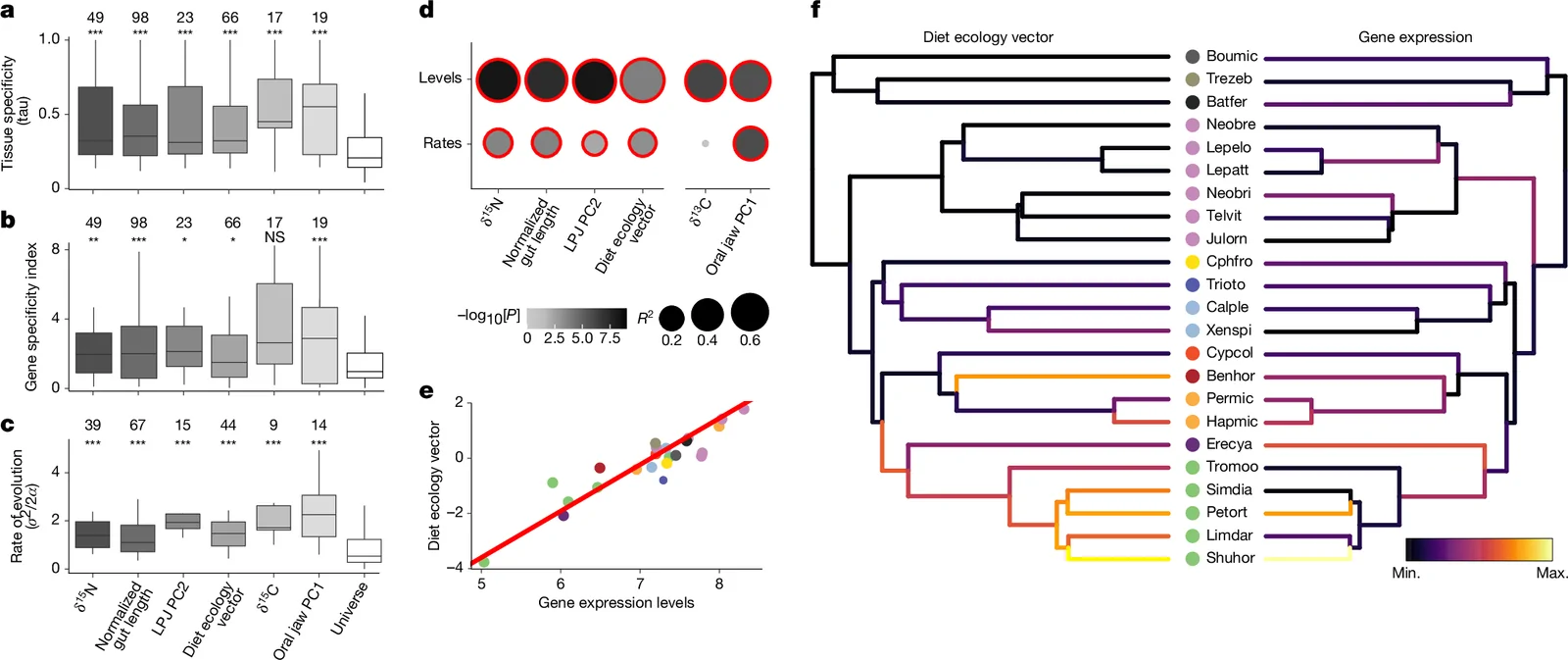
Gene expression evolution of diet-related genes in anterior enterocytes. **a**–**c**, Tissue specificity (tau) scores (**a**), gene specificity indices (**b**) and rate of gene expression evolution measured as stationary variance ($\frac{{\sigma }^{2}}{2\alpha }$) inferred from an OU process (**c**), for genes whose expression in enterocytes 1 is associated with each ecological predictor shown below. Corresponding numbers of genes are indicated above. In **c**, only genes best fitted by an OU model of continuous trait evolution are considered. The boxes in **a**–c show interquartile range, whiskers extend to values within 1.5 × interquartile range and horizontal lines mark the median values. Statistical difference between the ‘Universe’ gene set and the other gene sets was assessed by two-sided Wilcoxon rank-sum tests. ***P < 0.001, **P < 0.01, *P < 0.05. **d**, Top, summary dot plot of phylogenetic regressions (pGLS) between six ecological predictor values and species-specific gene expression levels of ecologically relevant genes. Bottom, summary dot plot of linear model correlations between the branch-specific evolutionary rates of the six ecological predictors and the branch-specific evolutionary rates of gene expression of ecologically relevant genes, across the phylogeny. Statistically significant correlations (*P* < 0.05) are indicated with a red outline. **e**, pGLS analyses showing strong, significant correlation between the diet ecology vector and mean species-specific expression levels of genes associated with the diet ecology vector (*P* < 1 × 10^−5^, *R*^2^ = 0.85, *λ* = 0). **f**, Time-calibrated species tree with branches coloured according to the branch-specific evolutionary rates for the diet ecology vector (left), and for the expression of genes associated with the diet ecology vector (right). The colours of the data points in **e** and **f** refer to the cichlid sub-clades (tribes) found in Lake Tanganyika, as in ref. ^5^ and shown in Extended Data Fig. 7a. Max., maximum; Min., minimum; NS, not significant.

**Extended Data Fig. 9:**
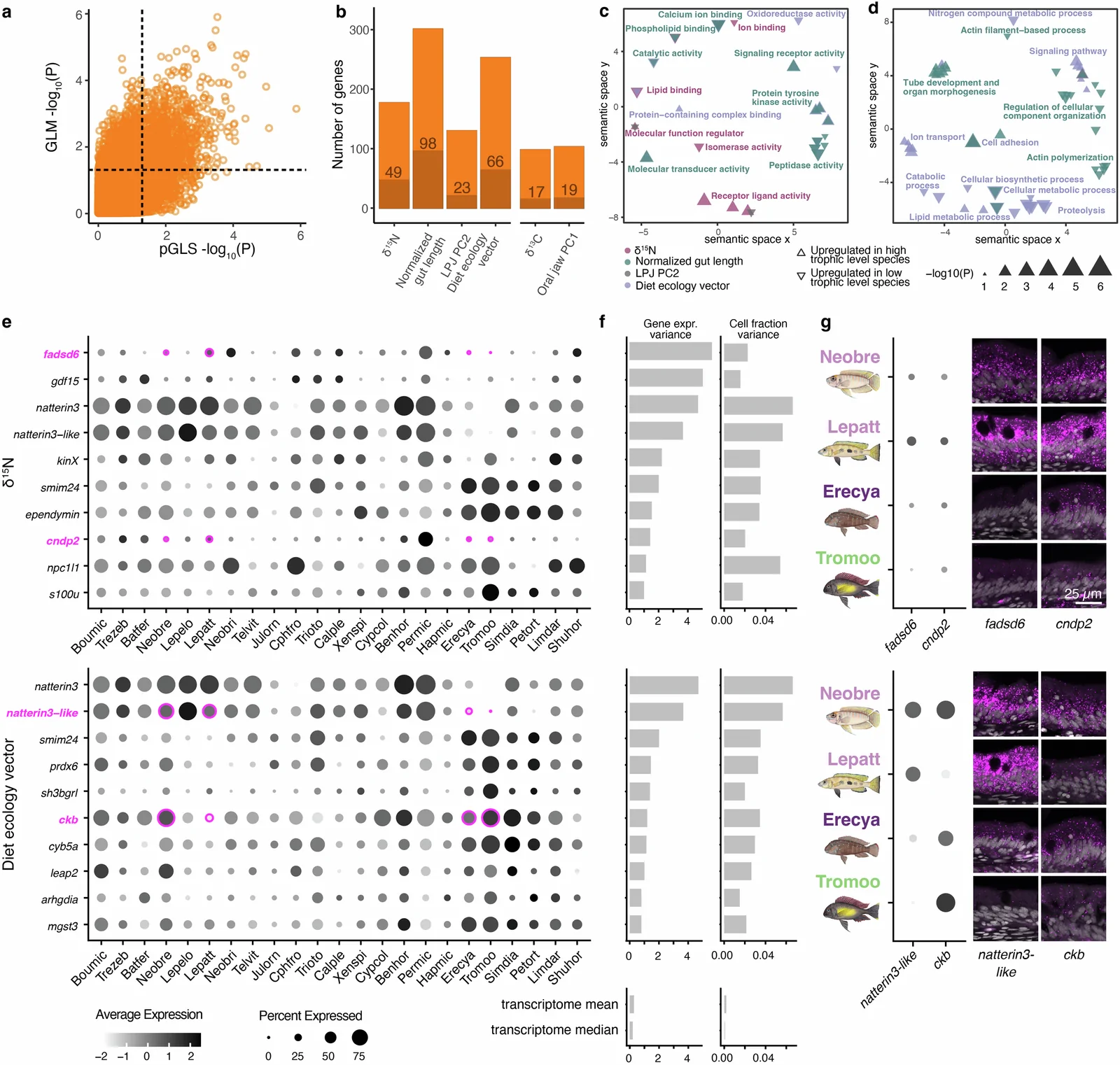
Detection and characterization of ecologically-relevant genes in anterior enterocytes. **a**, Distributions of P-values from lm and pGLS analyses, measuring the strength of association between gene expression and ecological predictors in enterocytes 1. **b**, Intersection of the four approaches used for finding genes whose gene expression correlates with ecological predictors. Lighter orange represents the number of genes identified through univariate approaches, and darker orange the strong candidate genes, identified as intersections of univariate and multivariate approaches. Numbers indicated on top of darker orange bars refer to the number of strong candidates detected. **c**, REVIGO scatterplot representation of Molecular Function GO terms enriched in genes expressed in enterocytes 1 and whose expression is associated with ecological predictors. **d**, REVIGO scatterplot representation of Biological Process GO terms enriched in genes expressed in enterocytes 1 and whose expression is associated with ecological predictors. In panels **c** and **d**, the relative sizes of the GO terms represent the P-values derived from GO-term enrichment analyses. **e**, Dot plot of expression of ten genes whose expression is associated with δ^15^N values (top) and of ten genes whose expression is associated with diet ecology vector values (bottom). **f**, Variance in gene expression levels (left) and in percentage of cells expressing genes (right) for genes from panel (e). The transcriptome mean and median variances of all genes expressed in enterocytes 1 is shown at the bottom of the panel. **g**, Dot plot of expression (left) and fluorescent in situ hybridization in anterior intestinal sections (right) of two genes whose expression are associated with δ^15^N values (top) and of two genes whose expression are associated with diet ecology vector values (bottom), in four ecologically diverse cichlid species. Images are representative of two independent experiments, using at least duplicate biological samples for each species. 25 µm scale bar applies to all images. Fish illustrations in** g** reproduced with permission from Julie Johnson.

**Extended Data Fig. 10:**
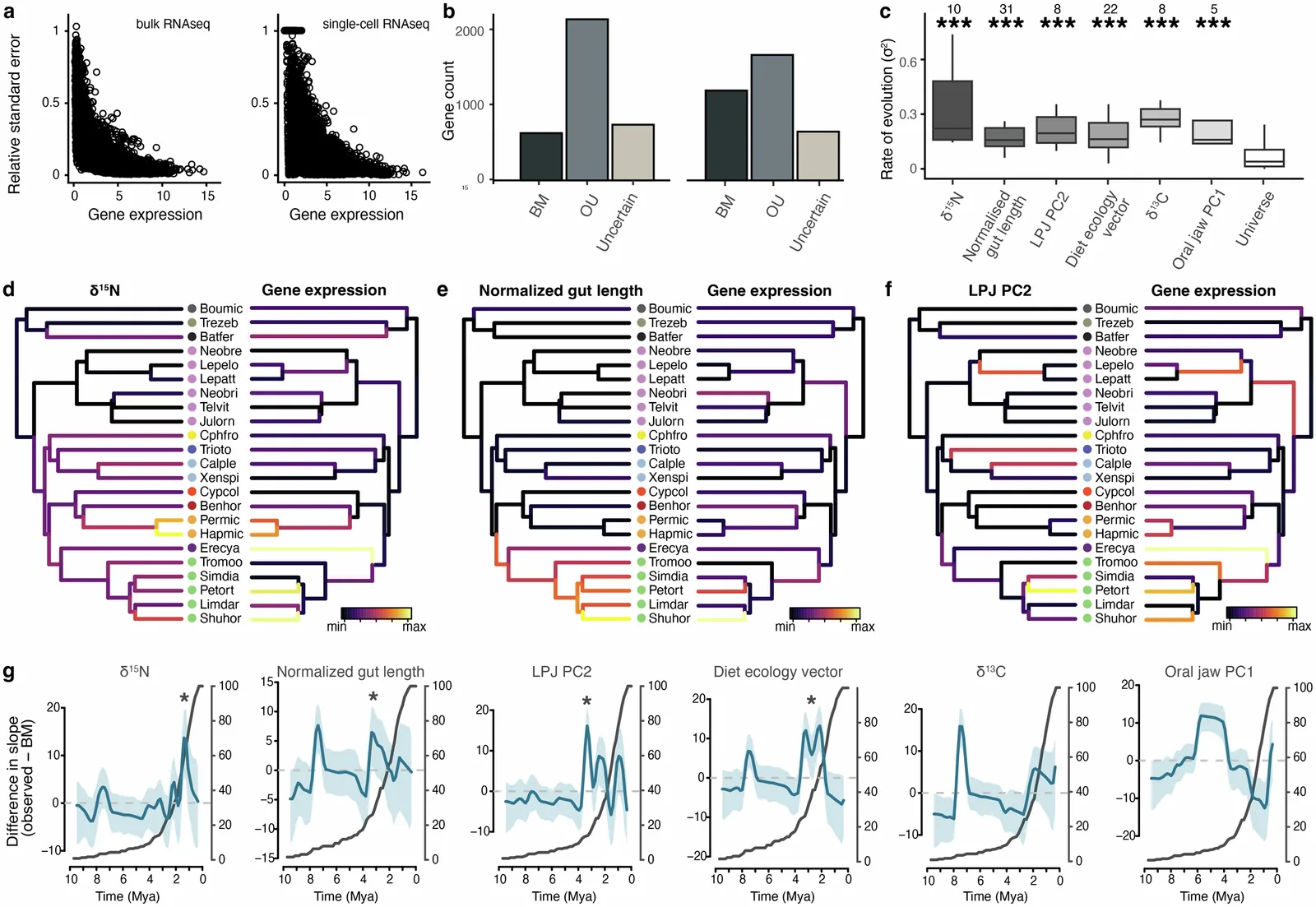
Reconstruction of gene expression evolutionary rates of ecologically-relevant genes in anterior enterocytes. **a**, Intraspecific variation as relative standard error, inferred from foregut bulkRNA-seq data (left) and from scRNA-seq pseudobulk data from enterocytes 1 (right). **b**, Best model of evolution for each gene, with intraspecific variation inferred from foregut bulk RNA-seq data (left), and from scRNA-seq pseudobulk data from enterocytes 1 (right). BM: Brownian Motion, OU: Ornstein Uhlenbeck, Uncertain: similar fit of both models, defined by |ΔAIC | <2 (ref. ^96^). **c**, Rate of gene expression evolution (σ^2^) inferred from a Brownian Motion (BM) process for genes whose expression is associated with ecological predictors in enterocytes 1 and for which a Ornstein-Uhlenbeck model is not a better predictor of gene expression evolution than a BM model. For each ecological predictor, the number of ecologically-relevant genes is indicated on top of the corresponding box plot. Boxes display interquartile range, whiskers extend to values within 1.5 interquartile range, and horizontal lines mark the median values. Statistical difference between the ‘Universe’ gene set and the other gene sets was assessed by two-sided Wilcoxon rank-sum tests. ***: P < 0.001, **: P < 0.01, *: P < 0.05, N.S.: non-significance (δ^15^N: P = 1.2e^−5^, normalized gut length: P = 3.0e^−10^, LPJ PC2: P = 3.0e^−4^, diet ecology vector: P = 2.7e^−7^, δ^13^C: P = 4.1e^−5^, oral jaw PC1: P = 4.1e^−3^). **d**, Time-calibrated species tree with branches colored according to the mean relative rates of trait evolution for δ^15^N (left), and for expression of genes associated with δ^15^N (right). **e**, Time-calibrated species tree with branches colored according to the mean relative rates of trait evolution for normalized gut length (left), and for expression of genes associated with normalized gut length (right). **f**, Time-calibrated species tree with branches colored according to the mean relative rates of trait evolution for PC2 of LPJ morphology (left), and for expression of genes associated with PC2 of LPJ morphology (right). **g**, Comparison of slopes (blue) of ecological space expansion over time for the six ecological predictors, between the observed data and the Brownian motion null model of trait evolution (mean across 500 Brownian motion simulations with 95% quantiles). Lineage accumulation through time derived from the species tree is shown in dark gray.

To uncover the molecular underpinnings of intestinal adaptations at the cellular level, we identified genes showing strong expression associations with ecological predictors in enterocytes 1, the cell population with the strongest ecological signals in both relative abundance and gene expression (Extended Data Fig. 9a,b). Consistent with these pronounced diet-related signatures, we found between 23 and 98 genes associated with each dietary predictor (δ^15^N values, normalized gut length, PC2 of LPJ morphology and diet ecology vector; Extended Data Fig. 9b). Interestingly, we also detected 17 genes correlating with δ^13^C values, probably due to the interdependence of nitrogen and carbon stable isotope signatures, as certain food sources are restricted to specific macrohabitats. These ecologically relevant genes showed high interspecific variability in expression (Extended Data Fig. 9e,f), which we further confirmed by ISH for four representative genes—two correlating with δ^15^N values and two with the diet ecology vector—in four ecologically distinct species (Extended Data Fig. 9g). We then assessed the tissue and cell population specificities of these genes by comparing them with all expressed genes in enterocytes 1, regardless of their ecological relevance (referred to as ‘Universe’ in Fig. 4a–c). Genes correlating with one or several ecological predictors exhibited higher tissue specificity (Fig. 4a; two-sided Wilcoxon rank-sum test, *P* < 0.001) and gene specificity indices (Fig. 4b; two-sided Wilcoxon rank-sum test, *P* < 0.05 for 5 of 6 gene sets) than the average of all expressed genes (‘Universe’). These findings indicate reduced pleiotropy and a higher potential for adaptive expression changes^41^ for genes of ecological relevance in enterocytes 1. We next investigated whether these gene specificities were associated with elevated rates of gene expression evolution. To this end, we modelled gene expression dynamics using either an OU or a Brownian motion (BM) process and estimated evolutionary rates for each expressed gene based on model parameters (Extended Data Fig. 10a,b). Consistent with previous findings^21^, the evolution of the expression of most genes was better captured by an OU model than by BM (Extended Data Fig. 10b). Most genes identified as ecologically relevant (Extended Data Fig. 9b) also evolved under an OU process and exhibited higher evolutionary rates than other expressed genes (Fig. 4c and Extended Data Fig. 10c; two-sided Wilcoxon rank-sum tests, *P* < 0.001), while having a similar phylogenetic age (two-sided Wilcoxon rank-sum tests, *P* > 0.05). This suggests that regulatory evolution of these genes in enterocytes 1 has been both adaptive and exceptionally fast, providing the substrate for functional and molecular specializations that enabled cichlids to rapidly diversify into distinct ecological niches. Moreover, these genes seemed enriched in GO terms associated with breakdown and absorption of fats and proteins in species at higher trophic levels, and with epithelial cell maintenance and ion balance in species at lower trophic levels (Extended Data Fig. 9c,d), suggesting a strong connection between gene function, expression levels and adaptations to nutrient absorption and uptake.

Finally, we explored how these genes may have contributed to the rapid ecological diversification of cichlid fishes in Lake Tanganyika. We first assessed the strength of association between various ecological predictors and species-specific mean gene expression levels across each gene set. Unsurprisingly, we identified strong associations for all ecological predictors (Fig. 4d, top). For example, we found that enterocyte 1 gene expression profiles correlate strongly with the diet ecology vector (Fig. 4d,e; lm and pGLS: *R*^2^ = 0.85, *P* < 1 × 10^−5^, *λ* = 0). We next inferred evolutionary rates for the different ecological predictors and for gene expression along the species phylogeny, using data from 126 to 234 cichlid species in Lake Tanganyika for ecological predictors and scRNA-seq data from 23 species for gene expression (Fig. 4d, bottom). These analyses again revealed strong correlations between dietary predictors and corresponding gene sets (lm: *R*^2^ > 0.2), despite the stark differences in species numbers and phylogenetic sampling density used for evolutionary rate inferences. More importantly, we detected heterogeneous rates of evolution across the radiation and identified bursts of accelerated evolution in both gene expression and ecological predictors, in particular in the Tropheini, a tribe known for numerous dietary transitions between algae browsing, algae grazing and even piscivory^42^ (green dots in Fig. 4f and Extended Data Fig. 10d–g). Notably, most ecologically relevant genes were best described by a two-optima OU model, consistent with an adaptive gene expression shift associated with herbivory (Methods and Supplementary Table 6). Our trait evolutionary analysis also confirmed previously identified pulses of ecological diversification in the adaptive radiation of cichlid fishes in Lake Tanganyika^5^, including a major pulse of dietary diversification around 1.5–4 million years ago (Extended Data Fig. 10g, asterisks; δ^15^N-values, normalized gut length, PC2 of LPJ morphology and diet ecology vector), which probably reflects fine-scale ecological niche partitioning through trophic specialization^5^.

Collectively, our findings strongly suggest that cellular adaptations arising from interspecific differences in gene expression within anterior-enriched enterocytes have facilitated feeding specializations, leading to rapid trophic niche partitioning and accelerated species diversification in the later stages of the adaptive radiation of cichlid fishes in Lake Tanganyika.

## Discussion

In this study, we generated extensive scRNA-seq data from the intestines of 24 ecologically distinct species of cichlid fishes from Lake Tanganyika, to investigate the cellular and molecular basis of dietary adaptations in the intestines of these rapidly evolving species (Fig. 1). By combining our multi-species single-cell gene expression data with comprehensive eco-morphological, genomic and bulk-transcriptomic information, we found the strongest signals of dietary adaptations in anterior-enriched enterocytes 1, especially with respect to relative cellular abundances and gene expression profiles. Interestingly, this enterocyte cell population is transcriptionally most similar to mouse duodenal enterocytes^18^, which have been shown to mediate lipid metabolism^15^ and to exhibit rapid plastic responses to novel food sources^43,44^. In cichlids, enterocytes 1 are overrepresented in carnivorous species compared with primary consumers, which in turn feature the highest relative proportions of secretory cells (Fig. 3b,c). Our results thereby align with previous feeding experiments in other animals, revealing changes in absorptive cell numbers resulting from high-fat or high-carbohydrate diets^44,45^. Importantly, however, we detect these compositional tissue changes in an evolutionary-comparative and ecological context, thus reflecting distinct physiological requirements associated with trophic specializations: carnivorous species at higher trophic levels, feeding on fat-rich diets, rely on a more efficient lipid metabolism and nutrient absorption along their shorter intestines, which may be facilitated by an increased relative abundance of enterocytes 1. As both absorptive and secretory cell populations in the intestinal epithelium originate from a common pool of stem cells, our results indicate that epithelial cell fate specification^46^ and/or progenitor pool expansion^47^ are ecologically tuned in cichlids. Although ontogenetic series would be required to determine the underlying molecular and temporal dynamics, our results suggest that, at least in adult fish, shifting the ratio between absorptive versus secretory cells is a major axis of rapid adaptation and specialization to diverse feeding habits. The extents to which these cell specification processes—and the intestinal architecture and function overall—are also influenced by varying intestinal microbial landscapes or immune system activities should be the subject of future research.

At the molecular level, we found strong associations between gene expression signatures in anterior enterocytes and our dietary predictors, largely driven by cell-population-specific and rapidly evolving genes (Fig. 4 and Extended Data Figs. 9 and 10). Interestingly, these enterocytes are also the intestinal cells with the highest overall gene expression divergence across species (Fig. 2a,b) and the ones with the weakest evolutionary and pleiotropic constraints acting upon their transcriptome (Fig. 2c–f), all of which have previously been linked to adaptive evolution^48,49^. Rates of gene expression evolution of diet-associated genes in anterior enterocytes were particularly elevated in branches of the species phylogeny for which rapid trophic shifts have been documented, especially in the later phase of the cichlid adaptive radiation in Lake Tanganyika^5,14,42,50^ (Fig. 4c–f and Extended Data Fig. 10d–g).

In summary, we provide evidence that trophic specializations in the digestive tract of cichlid fishes have occurred across multiple layers of biological organization, ranging from macro-morphological changes to cellular and molecular adaptations at the tissue composition and transcriptome levels of their intestines. Furthermore, our study demonstrates the power of integrating single-cell transcriptomics with morphological and ecological data, to help to uncover the complex cellular and molecular underpinnings of phenotypic evolution and organismal diversification within the context of adaptive radiation and beyond.

## Methods

### Sampling

All animals included in this study were kept in the fish facility of the Zoological Institute, University of Basel, Switzerland, under the experimental animal facility permit 1010H issued by the Cantonal Veterinary Office Basel Stadt, at a 12-h/12-h light/dark cycle at 25 °C. The different cichlid species were fed with different types of commercially available (cichlid) fish food to broadly match their natural diet category. Specific sample information (species, age and sex) can be found in Supplementary Table 1. Single biological replicates were sampled for each species, with no randomization or blinding. Animals were systematically sampled in the morning, at least 12 h after the last food intake, to optimize subsequent cleaning of the intestine. In brief, all individuals were euthanized with an overdose of KoiMed Sleep, followed by an incision in the spinal cord, in accordance with experimental permit 2317 of the Cantonal Veterinary Office Basel Stadt. The intestine of killed animals was then dissected, placed in ice-cold PBS and stored on ice until tissue dissociation.

### Generation of scRNA-seq data

Individual intestines were carefully flushed with ice-cold PBS under binoculars to remove all visible gut content, and cut into three equally long parts along the anterior–posterior axis, which were then dissociated individually. All intestinal samples (*n* = 75) were thoroughly chopped with a razor blade and dissociated at 25 °C for 20 min with low agitation (100 rpm) in either 1 or 3 ml of pre-warmed DMEM/F-12 medium 1X (Fisher Scientific) supplemented with 1 mg ml^−1^ Dispase II (Sigma). To ensure optimal dissociation of the tissue, samples were mixed by pipetting every 4 min throughout the enzymatic dissociation process. Suspensions were centrifuged at 500*g* for 7 min at 4 °C, resuspended in 10% fetal bovine serum (FBS) in DMEM/F-12, filtered with a 20-µm cell strainer (CellTrics), centrifuged again with the same conditions, resuspended in 10% FBS in DMEM/F-12 and centrifuged one last time at low speed (200–250*g*) for 5 min at 4 °C. Depending on the size of each sample, the pellet was resuspended in 100–1,000 µl of ice-cold 10% FBS in DMEM/F-12. Cell concentration and viability were estimated on a Cellometer K2 (Nexcelom). Cell viability was higher than 80% in all samples. For each intestine, the three independent gut samples were subsequently pooled, ensuring that each sample contributed equally to the final pool in number of cells.

All intestine cell suspensions were then diluted to a concentration of 500–1,500 cells per µl and loaded in a 10X Genomics Chromium for subsequent generation of single-cell complementary DNA (Chromium Next GEM Single Cell 3′ kit, 10X Genomics). Following the manufacturer’s instructions, dual index libraries were prepared and quantified either on a 2100 Bioanalyzer system (Agilent) or on a Tapestation 4200 system (Agilent). Libraries were sequenced on a NovaSeq 6000 system (Illumina) at the Genomics Facility Basel, jointly operated by the University of Basel and the Department of Biosystems Science and Engineering of ETH Zürich. A total of 61–297 million reads (median 117 million reads) were sequenced for each library.

### Generation of bulk sequencing data

Six individuals from three cichlid species (*Astatotilapia burtoni*,* Eretmodus cyanostictus* and *Lepidiolamprologus attenuatus*) were killed and dissected as described above. Cleaning of the intestines was also performed as described above. Each intestine was divided into five equally long parts along the anterior–posterior axis, and each section was processed independently. The sections were first mechanically chopped with a razor blade, then homogenized on a FastPrep 24 instrument (MP BioMedicals), and total RNA was extracted using a Zymo Direct-Zol RNA Miniprep kit (Zymo Research), following the manufacturer’s protocol. Illumina Truseq libraries (Illumina) were subsequently prepared and sequenced on a NovaSeq 6000 instrument (Illumina) at the joint Genomics Facility Basel, resulting in 11.4–17.3 million reads per sample.

### Raw scRNA-seq data processing

The software Cell Ranger v.5.0 (10X Genomics) was used to perform base calling, adaptor trimming and mapping the sequencing reads to the genome of the Nile tilapia (*O. niloticus*, GCA_001858045.3, RefSeq assembly GCF_001858045.2 (ref. ^51^)), a closely related cichlid species that is phylogenetically equidistant to all endemic Tanganyikan cichlid species included in this study, and for which high-quality chromosome-level assembly and annotation are publicly available. Between 46.4% and 86.2% of sequencing reads (mean 67.5%) mapped confidently to the reference genome, and 32.5–75.6% (mean 52.8%) mapped confidently to the reference transcriptome (Supplementary Table 2). We obtained a cell/gene count matrix for each sample, which consisted of 1,133–8,226 cells (mean 4,498 cells), with a mean of 33,058 reads, 2,255 unique molecular identifier (UMI) counts and 713 detected genes (Extended Data Fig. 1a–c). In total, we detected in each sample between 20,877 and 26,535 genes (mean 24,195 genes).

These cell/gene count matrices were filtered to eliminate low-quality cells. In short, we first corrected the gene expression data for ambient mRNA contamination based on the estimated mRNA expression profile from empty droplets, using the R package SoupX^52^ (v.1.6.2). We filtered out all cells with a mitochondrial contribution to UMI count higher than 80% and cells with a low ratio of detected genes per UMI counts. We followed the filtering procedure implemented in the R package ddqcR^53^ (v.0.1), which filters out cells with fewer than 100 detected genes, clusters the data into biologically meaningful clusters and performs a data-driven quality control at the level of clusters, using three successive filtering steps: (1) keeping cells that have UMI counts greater than median value −2 median absolute deviations (MAD), (2) retaining cells that have a number of detected genes greater than median value −2 MAD, and (3) keeping cells that have a fraction of reads mapping to mitochondrial genes less than median value +2 MAD.

Subsequently, we performed normalization of the gene expression data from all retained cells of each sample and cell clustering using the R package Seurat^54^ (v.4.1.0). Specifically, we first used the SCTransform function^55^ to normalize the data, using the fraction of UMI counts mapping to mitochondrial genes and the fraction of UMI counts mapping to ribosomal genes as regression variables. We then performed a PCA using the RunPCA function of the R package Seurat, and the cells of each sample were clustered based on the first 30 components of the PCA using the FindNeighbors and FindClusters functions, with default parameters and a resolution of 1.

### Integration of single-cell datasets

All single-cell clustered and normalized datasets were integrated using the SelectIntegrationFeatures, PrepSCTIntegration, FindIntegrationAnchors and IntegrateData functions from the R package Seurat^54,56^ (v.4.1.0) with 5,000 features and a reference-based integration approach. The resulting integrated dataset was normalized and scaled, using the regression variables mentioned above, and a cell cycle score computed using the CellCycleScoring function and a database of 87 Nile tilapia orthologues of zebrafish genes known to be involved in cell cycle. The resulting dataset was clustered based on the top 100 PCA components and the Louvain algorithm with a resolution of 3, and visualized with the UMAP algorithm, using the RunUMAP function. We then filtered out non-informative clusters with a median combined mitochondrial or ribosomal fraction higher than 50%, performed an extra clustering step with similar parameters and obtained a final integrated dataset composed of 82,621 high-quality clustered cells derived from 25 species (Fig. 1b).

### Identification of biological cell types

Following the initial clustering, we identified all major clusters as cell type categories (Fig. 1b,c). Cell population characterizations were performed within each cell type category, using a finer sub-clustering with low resolution parameters (0.1–0.4). Cell types were identified based on the expression of cell type marker genes described in the literature, and by contrasting uncorrected gene expression patterns between clusters, as performed in the FindAllMarkers function from the R package Seurat, with the following parameters: min.pct = 0.25, logfc.threshold = 0.5, method = ‘MAST’ (Fig. 1c, Extended Data Fig. 1g,h and Supplementary Table 3). Overall, we identified nine epithelial cell populations, including four absorptive and five non-absorptive cell populations, one of which we classified as ‘proliferating secretory cells’ on the basis of its expression of cell cycle and pan-non-absorptive cell marker genes, as well as its clustering with other secretory cell populations inferred from global gene expression profiles (Fig. 1d,e and Extended Data Fig. 1f,i,j). Whether this cell population could serve as an epithelial progenitor pool, to also give rise to differentiated absorptive cells, would require further work, including detailed ontogenetic timeseries and potential lineage tracing. Subsequently, cross-species identification of cell types was validated using functions from the R packages SciBet^57^ (v.1.0) and MetaNeighbor^58^ (v.1.14) (Extended Data Fig. 1e). In brief, the MetaNeighborUS function was used to compute cell type to cell type area under the receiver operator characteristic curve (AUROC) scores and verify that identified cell types were robust to cross-species differences. The topHits function was then called with a threshold parameter of 0.8 to visually inspect for potential high matches between different cell types. The conf function from the R package SciBet was used to generate a confusion matrix that evaluates the performance of the cell type classification, to ensure that diagonal values, which correspond to self-validation scores, were higher than 0.8. To further characterize the identified cell types, we estimated the fraction of reads mapping to mitochondrial genes and counted the number of counts (UMIs) and detected genes in each cell of each cell type (Extended Data Fig. 1a–c). Finally, for each cell type, we calculated the proportions of transcripts that show high cell population specificity in expression, as defined by a gene specificity index higher than 6. This revealed a variable number of cell-population-specific features among cell types (Extended Data Fig. 1d).

### Characterization of epithelial cells

We identified four main populations of enterocytes in our single-cell datasets, which we further characterized by examining the expression of 1-to-1 orthologues of zebrafish genes that show conserved regional transcriptional specification along the intestine in zebrafish and mice^18,59,60^ (Extended Data Fig. 3a). This analysis revealed strong anterior–posterior signatures for enterocytes 1, and, to a lesser extent, in enterocytes 2 and LREs, as previously shown in the zebrafish^16,61^.

To confirm these findings, we applied spatially defined bulk mRNA sequencing from three species included in the single-cell dataset (see the section ‘Generation of bulk sequencing data’). The obtained sequencing reads were trimmed using Trimmomatic^62^ (v.0.39), and mapped to the genome of the Nile tilapia using STAR^63^ (v.2.7.3) and HTSeq^64^ (v.2.0) (Supplementary Table 4). We then used the R package BayesPrism^19^ (v.2.0) to deconvolute the bulk in each part of each species separately, and to predict cellular composition in cell types from total RNA-seq data, using both mRNA and single-cell raw counts as input (Extended Data Fig. 3b,c). This revealed strong anterior–posterior identity in enterocytes 1, enterocytes 2 and LREs. In our deconvoluted bulk RNA-seq data, enterocytes and secretory cells seem to make up the most of the cell population proportions. This is probably due to the higher levels of expression in these cell populations, compared with non-epithelial cells, whose transcriptomic signatures may not be adequately captured in bulk RNA-seq, and with biases in tissue dissociation and isolation of single cells for scRNA-seq, in which non-epithelial cells may be preferentially dissociated and isolated owing to less pronounced cell–cell adhesion.

To characterize the cellular and molecular functions inherent to the three major types of enterocytes, we calculated pseudobulk values for each gene in each cell type and contrasted the gene expression signatures through differential expression analysis. Finally, we performed GO^65^ term enrichment analyses on the sets of differentially expressed genes using the R packages limma^66^ (v.3.50.3) and GO.db (v.3.14), and visualized these using the REVIGO web server^67^. This revealed the predicted molecular functions, cellular components and biological processes associated with each type of enterocyte and, by extension, with each part of the intestine (Extended Data Fig. 3d–g).

To experimentally validate the anterior–posterior identity of enterocytes in the cichlid intestine, we conducted fluorescent ISH experiments for marker genes of enterocytes 1 (*amcase-1*) and LREs (*cubn*). In brief, we cloned antisense probes from Nile tilapia intestinal cDNA using PCR primers designed based on bulk RNA-seq expression profiles. Details about the primer pairs can be found in Supplementary Table 5. For tissue sampling, we dissected, flushed and paraformaldehyde-fixed intestine sections from four cichlid species, at five equidistant positions along the anterior–posterior axis. Tissue of all four species, and all five gut regions, was cryo-embedded, sectioned and processed together, to ensure similar staining conditions throughout the procedure. We followed standard hybridization protocols, with fluorescent signal amplification using TSA Plus Cyanine-3 or -5 (Akoya Biosciences)^68,69^. All fluorescent images were acquired on an Olympus FLUOVIEW FV3000 confocal microscope (Extended Data Fig. 4).

### Characterization of gene expression signatures

We first assessed gene expression affinities between cell populations by calculating Pearson pairwise correlations between cell populations and a hierarchical cluster analysis based on the ward D2 agglomeration method (Fig. 1d). In addition, for each cell type and species, we generated pseudobulk samples by aggregating raw counts across cells, using the GetAssayData function from the R package Seurat. We filtered out from the intestine matrix of pseudobulk samples lowly expressed genes that are not expressed in at least two cells of any cell type, across all species. The resulting data consisted of 13,150 genes, whose expression levels were quantified in all 24 identified cell populations.

To visualize global patterns of gene expression differences across species and cell populations, the gene expression data were further normalized using the median of ratios method implemented in the R package DESeq2 (ref. ^70^) (v.1.14) to account for between-sample differences in sequencing depth and RNA composition. We next transformed the data using the variance-stabilizing transformation method as implemented by the vst function from DESeq2 and performed a PCA based on the top 10,000 variable genes using the prcomp function (Fig. 1e and Extended Data Fig. 1i,j).

Subsequently, the pseudobulk expression values were normalized to counts per million (cpm) values, which were used for all downstream analyses. To account for potential differences and biases in expression quantification across species and single-cell data generation batches, we calculated a gene specificity index for each gene, species and cell type, as defined in ref. ^71^ (Extended Data Fig. 1f). In short, for every species included, we computed for each gene the ratio between its level of expression within each cell type and its mean expression across all cell types and performed pairwise correlations across all species and cell types, using Spearman’s rank correlation coefficients.

### Cross-species gene expression divergence

We calculated cross-species gene expression in a pairwise fashion and within each cell population, after filtering out pseudobulks aggregated on fewer than 20 cells, and cell populations with fewer than 17 species represented by at least 20 cells. This resulted in a dataset consisting of cpm values calculated in 18 cell populations, with varying numbers of species represented in these cell populations. For each pair of species and within each cell population, we computed the mean-squared distances as implemented in EVEE-tools^24^ (Fig. 2a). To investigate whether gene expression divergence tends to correlate positively with phylogenetic divergence, we also retrieved published evolutionary distances between all pairs of species in the dataset, calculated as genomic substitutions across the genome^5^, and plotted gene expression mean-squared distances as a function of evolutionary distances, revealing a nonlinear, positive correlation between them (Extended Data Fig. 5). Specifically, following work in ref. ^24^, we observed that the interspecific gene expression distance (*y*) seems to evolve as a function of the interspecific genomic distance (*x*) in power law fashion (*y* = *ax*^*k*^), which is an expected relationship under an OU model of evolution^24^. We therefore fitted both this model and a linear model (which would be expected under a BM mode of gene expression evolution) to our empirical data, and show that based on the Akaike information criterion (AIC), the power law model is a better fit than the linear one in all cell populations, except in dendritic cells, T cells 1 and T cells 3.

Cross-species gene expression divergence was also estimated with the R package Augur^28^ (v.1.0.3), using a method designed for prioritizing cell type responses to experimental conditions, with conditions set to ‘species’ here. In short, Augur tries to predict which species each cell from a given cell type comes from, based on gene expression, and gives an overall AUC, a confidence score that ranges from 0.5, if the species identity cannot be recovered better than randomly, to 1, if the species identity is always correctly guessed.

To account for potential differences in sequencing depth, each cell was downsampled to the same number of UMIs (1,000 UMIs per cell). The Augur method was applied to the integrated dataset restricted to the 18 selected cell populations with enough species represented by 20 cells or more. Augur was then run in a pairwise fashion, in subsets of the dataset containing only two of the species, with the following parameters: subsample_size = 10, min_cells = 10, n_subsamples = 50. Pairwise AUC scores were compared across the 18 selected cell populations, as well as across pairs of species and phylogenetic distances separating these pairs (Fig. 2b).

### Cell-type-specific transcriptomes

We used four different approaches to characterize the genes expressed in each cell population, and the evolutionary forces at play that may constrain the evolution of cell populations and of their associated transcriptomes. First, we reconstructed the phylogenetic age index of each gene in the genome of the Nile tilapia, calculated as phylostrata values, whereby younger genes exhibit higher values and older genes lower ones. To do so, we followed the procedure implemented in the Python package oggmap^72^ (v.0.0.1), which relies on a phylostratigraphic approach to extract gene ages in orthology groups, enabling us to calculate the phylogenetic ages for 12,550 such orthologues expressed in our intestine single-cell dataset (Fig. 2c), which we then used to estimate the TAI of each cell.

Second, we computed tissue specificity indices (tau), a measure of how tissue-specific the expression of a gene is, which ranges from 0 to 1. We used the procedure from ref. ^21^, based on gene expression data derived from 13 tissues of cichlid fishes: brain, retina, gonads, LPJ, gills, liver, intestine, skin, spleen, kidney, heart, muscle and blood^21,73–78^. This resulted in tau values for 25,687 genes expressed in the intestine (Fig. 2d).

Third, we retrieved predicted PPIs in *O. niloticus*. PPIs have previously been linked to constrained evolution^79^ and have been used in the literature as a proxy for pleiotropy, with genes involved in a high number of PPIs typically being considered more pleiotropic^41,80^. We extracted PPIs from the STRING v.11 database^34^, and selected high-confidence PPIs, as indicated by a score higher than 0.7 (ref. ^81^), which resulted in high-confidence PPI predictions for a total of 13,977 PCGs expressed in the intestine (Fig. 2e).

Fourth, we retrieved from ref. ^35^ ratios of non-synonymous to synonymous substitution (d*N*/d*S*), calculated for 4,172 orthologous PCGs among three species of distantly related teleost fish species, namely, *Tetraodon nigroviridis*, *Takifugu rubripes* and *Danio rerio*. We used d*N*/d*S* as a proxy for the evolutionary selective pressure acting on PCGs. For each metric presented above and each cell present in the integrated dataset, we weighted each individual gene score by its expression value within the cell and computed the overall cell TAI, tau, PPI and d*N*/d*S*, by calculating the mean of these weighted scores across all genes expressed (that is, with UMI count > 1; Fig. 2f). For each one of the four metrics (TAI, tau, PPI, d*N*/d*S*), we statistically assessed differences across intestinal cell populations. To do so, we first calculated the mean value of each metric across all cells of each cell population and species and subsequently performed paired phylogenetic *t*-tests for each pair of cell populations (Extended Data Fig. 6a–d) and for each pair of cell type categories (Extended Data Fig. 6e–h), using the cell-population- (and cell-type-category-), species-specific mean values as paired variables.

### Detection of gene co-expression modules

We detected gene co-expression modules using the R package scWGCNA^40^, an adaptation of WGCNA^82^ for single-cell datasets. In total, we identified 25 modules of co-expressed genes, comprising 17–103 genes each (mean = 53 genes; Extended Data Fig. 2a). We next quantified module expression in each identified cell type to characterize the cell type specificity and molecular functions of each scWGCNA module. GO term enrichment analyses were then performed using the R packages limma and GO.db, and visualized using the REVIGO web server^67^, to highlight molecular functions associated with the modules of co-expressed genes (Extended Data Fig. 2b,c).

### Ecological predictors

We used a total of six eco-morphological proxies. First, we retrieved the stable carbon isotope (δ^13^C values) and stable nitrogen isotope (δ^15^N values) compositions of muscle tissue, previously measured for all cichlid species of the adaptive radiation of cichlid fishes in Lake Tanganyika^5^. δ^13^C values serve as a proxy for the position of species along the benthic–pelagic axis (with higher δ^13^C values being associated with macrohabitats close to the shore of the lake, and lower δ^13^C values with pelagic environments), whereas δ^15^N values are used as a proxy for trophic level, as primary consumers (typically herbivorous/algivorous species) have lower δ^15^N values than predatory species^5,83^. Subsequently, we retrieved gut length data across 126 species from ref. ^38^, and calculated the normalized gut length (so-called Zihler index^84^; Extended Data Fig. 7a), which has been shown to negatively correlate with trophic level in various fish taxa^38,85,86^, including cichlid fishes^50^. Additionally, we included two other morphological predictors in our analyses: PC2 of LPJ morphology and PC1 of oral jaw morphology, measured across virtually all species of the cichlid adaptive radiation in Lake Tanganyika^5^, which have been shown to be mostly associated with trophic ecology and food uptake, respectively. Finally, we reconstructed a diet ecology vector, representing PC1 of a PCA calculated from stable nitrogen isotope data, LPJ morphology and normalized gut length data (Extended Data Fig. 7b,c).

### Ecological signals in cell type abundances

For each specimen, we estimated the relative abundances of the different cell populations using the plotCellTypeProps function from the R package speckle^87^ (v.0.0.3). Because the efficiency of dissociation of epithelium and of the underlying layer of connective tissue (lamina propria) can vary from species to species, owing to biases in intestine thickness, robustness and elasticity, we focused on the epithelial cell types, which form a single layer of cells, separating the intestinal lumen from the lamina propria. Specifically, we calculated the relative abundance of all epithelial absorptive and non-absorptive cells within the epithelium (Fig. 3a). We next sought to test for association between relative cell type abundances and ecological predictors. First, to account for the intercorrelations between relative abundances, we transformed the cell population compositional data into centred log-ratios, and the overall relative abundances of absorptive cells using a logit transformation^88,89^, as implemented in the R package compositions^90^ (v.2.0.4). Finally, we applied a linear regression (lm function in R) and a phylogenetic generalized least-square analysis (pgls function from the R package caper^91^ (v.1.0.3)), which accounts for the phylogenetic non-independence of the species. We reported the coefficients of determination and *P* values adjusted for false discovery rate to identify cell types and cell categories whose differences in relative abundances are ecologically relevant (Fig. 3c).

### Quantification of expression levels of genes in the intestines of cichlid fishes

To experimentally validate our cell population abundance findings with an orthogonal method, we designed RT–qPCR primer pairs for six genes expressed at similar levels across species within cell populations: two genes expressed in absorptive cells but not in non-absorptive cells (*pdzk1* and *apob100*), two genes expressed in enterocytes 1 but not expressed in other epithelial cells (*amcase-1* and *i-fabp*), *epcam* as a pan-epithelial marker and *rpl7* as a housekeeping gene and endogenous control. Details about the primer pairs, including GC content, length and sequence, can be found in Supplementary Table 5. In short, we dissected whole intestines from four individuals of four cichlid species with distinct dietary ecologies and characterized by different δ^15^N values (the piscivorous *L. attenuatus*, the planktivorous *Neolamprologus brevis* and the algivorous *Tropheus moori* and *E. cyanostictus*), incubated them in RNAlater (Qiagen) overnight at 4 °C and extracted total RNA using a Direct-Zol RNA miniprep kit (Zymo). RNA was quantified on a Nanodrop instrument (ThermoFisher Scientific) and retrotranscribed into cDNA using a GoScript Reverse Transcription, oligo(dT) kit (Promega). The resulting cDNA was subjected to RT–qPCR in triplicates for each of the six primer pairs using PowerUp SYBR Green Mix (ThermoFisher Scientific) on a Quantstudio 3 Real-Time PCR system (Applied Biosystems), using 2 μl of 1:8 dilutions of cDNA as input and following the standard cycling mode. Using *epcam* and *rpl7* for reference and normalization, our analyses show that relative expression of absorptive genes and enterocytes 1 genes correlates with relative abundance of enterocytes 1, as inferred from our scRNA-seq data, thus confirming our findings (Extended Data Fig. 7d).

### Associations between ecology and cell populations

To investigate associations between gene expression in intestinal cell populations and ecological predictors, we created three sets of genes. For each cell population, we first selected all genes expressed in at least 50% of the species and in 20% of the cells, which we refer to as the ‘global’ gene set. In parallel, we established a ‘specific’ gene set, consisting only of cell-type-specific genes (as defined in the section ‘Identification of biological cell types’). Finally, we constructed a third ‘module’ gene set, consisting of the genes of scWGCNA modules only. We then used the cpm pseudobulk dataset presented in the section ‘Cross-species gene expression divergence’, consisting of 18 cell populations and with varying number of species per cell population, according to how many of these species are represented by at least 20 cells and by a sufficient number of counts in each cell population. This gene set was log_10_-transformed and filtered for each one of the three sets of genes, resulting in a ‘global’ gene set consisting of 18 cell populations and 454–3,221 genes (mean = 1,607 genes) per cell population and a ‘specific’ gene set, consisting of 18 cell populations and 30–360 genes (mean = 132 genes) per cell population. To build the ‘module’ gene set, for each module we selected the cell population in which the module expression was the highest, and filtered out three modules with either low cell population specificity or high specificity in a very rare cell population. This resulted in a ‘module’ gene set consisting of 22 gene expression matrices with 17–103 genes per matrix (mean = 53 genes).

For each one of the three gene sets, we assessed the association between each ecological predictor and cell population gene expression using a multivariate phylogenetic regression approach, as implemented in the functions mvgls and manova.gls of the R package mvMORPH^39,92,93^ (v.1.1.8). Specifically, we fitted a BM model of evolution with a Pagel’s lambda phylogenetic tree transformation and a penalized likelihood approach, and applied a type II phylogenetic multivariate analysis of variance using the Pillai’s test statistic and 1,000 permutations to detect potential significant correlations (Extended Data Fig. 8a–c).

To identify which ecological predictors are most relevant in ecology–gene expression correlations, we performed p2B-PLS analysis, as implemented in the two.b.pls function of the R package geomorph^94^ (v.4.0.7). The multivariate predictor matrix consisted of the nitrogen and carbon stable isotopes, as well as gut normalized gut length, PC2 of LPJ morphology and PC1 of oral jaw morphology. The individual ecological predictor loadings were then compared in each cell population of each one of the three datasets (Extended Data Fig. 8d–f).

### Characterizing ecologically relevant genes

To characterize associations between ecological predictors and gene expression profiles, we investigated correlations at the individual gene level in cell populations. Within each epithelial cell population, we conducted linear regression (lm) and pGLS analyses between each individual ecological predictor and all individual genes expressed in at least 10% of cells and across at least 50% of species. Specifically, we tested for correlations between the predictor values and the log_10_-transformed cpm pseudobulk values, using the time-calibrated genome-wide species tree of Tanganyikan cichlids to account for the non-phylogenetic independence of the species. For each ecological predictor, we selected the genes showing significant correlations in both lm and pGLS analyses (*P* < 0.05; Extended Data Fig. 9a).

Additionally, we performed a two-block partial least-squares (2B-PLS) analysis, as well as a p2B-PLS analysis, using as input each ecological predictor individually and the overall log-transformed filtered cpm pseudobulk gene expression matrix for the cell population enterocytes 1, that is, the cell population showing the most significant associations with dietary predictors, using the 2b.pls and phylo.integrations functions from the R package geomorph^94^. We then identified the top genes driving the correlations between the two blocks, selecting the same number of genes as those correlating with predictors at the individual levels in lm and pGLS analyses, and calculated the intersection between (1) the significant genes in lm and pGLS analyses, (2) the top 2B-PLS genes and (3) the top p2B-PLS genes, to create sets of genes correlating with ecological predictors at both the univariate and multivariate levels, and both with and without accounting for the phylogenetic non-independence of the samples during data rotation (Extended Data Fig. 9b).

We further characterized the ecologically relevant genes in enterocytes 1 by comparing them with all genes expressed in at least 10% of cells in each cell population in terms of tissue specificity (tau; Fig. 4a) and gene specificity index (Fig. 4b). We performed GO enrichment analysis to identify molecular functions and biological processes enriched in genes whose expression correlated positively and negatively with trophic levels, respectively. Finally, we visualized these using the REVIGO web server^67^ (Extended Data Fig. 9c,d).

For visualization of expression differences in ecologically relevant genes in an intact tissue context, we followed the same procedures for probe cloning, ISH and image acquisition as detailed above, for enterocytes 1 and LRE marker genes, the one exception being that we focused only on the anterior-most section of the gut, that is, where enterocytes 1 mostly reside (Extended Data Fig. 9g).

### Reconstruction of gene expression and trait evolution

For each cell population and expressed gene, we modelled the evolution of gene expression across the species phylogeny, as defined by the time-calibrated genome-wide phylogenetic tree^5^. To do so, we first excluded the outgroup Nile tilapia, and genes expressed in less than 50% of the species and in less than 10% of cells. We log-transformed the filtered cpm pseudobulk gene expression data and used the fitContinuous function from the R package geiger^95^ (v.2.0.7) to fit BM and OU models of evolution on each expressed gene individually. To account for intraspecific variation in gene expression modelling, we inferred the within-species relative standard error (RSE) (1) from bulk RNA-seq derived from our foregut bulk RNA-seq data (sections 1 and 2 along the anterior–posterior axis) and (2) from scRNA-seq enterocytes 1 pseudobulk data, using two biological duplicates generated from the intestines of two cichlid species, *Perissodus microlepis* (Permic) and *Cyphotilapia frontosa* (Cphfro). We found that the RSE evolves as a function of gene expression in a hyperbolic fashion: RSE = $\frac{a}{{\rm{Gene}}\,{\rm{expression}}+c}$, where *a* and *c* are two constants, here falling between 0.2 and 0.7 (Extended Data Fig. 10a). We then modelled intraspecific standard error based on the fitted hyperbolic function and used it as input in the SE parameter of the fitContinuous function. Finally, the fit of BM and OU models was compared using the AIC, and we selected for each gene the best model, defined by lowest ΔAIC and (|ΔAIC| > 2)^96^ (Extended Data Fig. 10b). Among genes best fit by an OU model, we next sought to test whether these were best modelled by a single-optimum OU, or by a two-optima OU, in which all herbivorous and/or algivorous Eretmodini and Tropheini species *Eretmodus cyanostictus* (Erecya), *Tropheus moorii* (Tromo), *Petrochromis orthognathus* (Petort) and *Simochromis diagramma* (Simdia) would evolve under a regimen distinct from the rest of the Lake Tanganyika cichlid phylogeny. To do so, we fitted a single-optimum OU model and a two-optima OU model using the OUwie function from the R package OUwie (v.2.10) and compared them using the corrected AIC (AICc). Individual gene expression evolutionary rates were inferred from the output of fitContinuous for each expressed gene in enterocytes 1 with intraspecific variation inferred from scRNA-seq enterocyte 1 pseudobulk data, based on model parameters from the best supported model: stationary variance, ($\frac{{\sigma }^{2}}{2\alpha }$), for genes best modelled by an OU model, and *σ*^2^ from the BM model for all other genes (Fig. 4c and Extended Data Fig. 10c).

To reconstruct the evolution of entire sets of ecologically relevant genes along the phylogeny of Tanganyikan cichlids and to identify lineage-specific rates of evolution, we first calculated the median value of gene expression for each set of genes (Fig. 4d). We next mapped the evolution of the median gene expression values onto the time-calibrated phylogenetic species tree (Fig. 4e,f) by modelling gene expression evolution as an OU process, the gene expression of the vast majority of ecologically relevant genes being best fitted by this model of trait evolution (141 out of 165 genes). Finally, internal node values were reconstructed using a maximum-likelihood approach, as implemented in the contMap function from the R package phytools^97^ (v.2.1.1), and the rates of gene expression evolution were visualized using a custom R script (Fig. 4f and Extended Data Fig. 10d–f).

Last, we estimated branch-specific rates for each ecological predictor—namely δ^15^N, δ^13^C, normalized gut length, PC2 of LPJ morphology, PC1 of oral jaw morphology and the diet ecology vector—by fitting a variable rates model using BayesTraits^98^ (v.3.0.2). For each trait we ran a single Markov chain Monte Carlo chain for at least 1,000,000,000 iterations, sampling parameters every 100,000 iterations after discarding the first 10,000,000 iterations as a burn-in. Stationary of each chain was accepted when the effective sample size of each parameter reached at least 200 (calculated using the R package coda^99^ (v.0.19.3)). For the parameter alpha (ancestral value) and the rate parameter sigma, we defined uniform prior distributions per trait (δ^13^C: alpha = −50–0; sigma = 0–10; δ^15^N: alpha = 0–20; sigma = 0–10; normalized gut length: alpha = −10–5; sigma = 0–20; diet ecology vector: alpha = −10–10; sigma = 0–10; LPJ PC2 and oral jaw PC1: alpha = −1–1; sigma = 0–0.001) that were informed from a single-process BM model, fitted using the R package geiger. For each ecological predictor, we then extracted branch-specific evolutionary rates from the mean rate-transformed tree of the posterior sample and reconstructed maximum-likelihood ancestral states using the R package phytools and compared it with the tree of median expression of ecologically relevant genes using a linear model (Fig. 4f and Extended Data Fig. 10d–g). To compare the diversity accumulation over time relative to a null model of trait evolution^5,100^, we further reconstructed rate-informed maximum-likelihood ancestral states using the R package phytools and calculated ecological diversity in time intervals of 0.15 million years (as in refs. ^5,14^), once from the ancestral states of the observed data and once based on ancestral states from 500 datasets that were simulated under BM. Finally, we compared the slopes of the diversity accumulation over time of observed data with each of the null models (observed–BM; Extended Data Fig. 10g).

### Reporting summary

Further information on research design is available in the Nature Portfolio Reporting Summary linked to this article.

## Online content

Any methods, additional references, Nature Portfolio reporting summaries, source data, extended data, supplementary information, acknowledgements, peer review information; details of author contributions and competing interests; and statements of data and code availability are available at https://doi.org/10.1038/s41586-026-10494-8.

## Supplementary information

Reporting Summary

Supplementary Table 1 General information about specimens and species included in this study.

Supplementary Table 2 Mapping statistics of single-cell RNA sequencing data per species.

Supplementary Table 3 Identification of genes specific to each cell population.

Supplementary Table 4 Mapping statistics of bulk RNA sequencing data per sample.

Supplementary Table 5 Information about primer pairs used for reverse transcription quantitative PCR (RT–qPCR) and in situ hybridization (ISH).

Supplementary Table 6 Total numbers of genes (non-ecologically relevant versus ecologically relevant) expressed in enterocytes 1 whose gene expression evolutions are best modelled by OU (row 3), sub-divided by whether a single-optimum (row 4) or a two-optima (row 5) OU model of trait evolution fits their expression dynamics best, or whether no clear trend could be observed (row 6). Intraspecific variation was incorporated in the model as inferred from foregut RNA-seq data (bulk scheme) or from enterocytes 1 pseudobulk derived from gut scRNA-seq data (pseudobulk scheme) (Extended Data Fig 10a).

## Data Availability

All single-cell and bulk RNA-seq raw and processed data generated in this study have been deposited at GEO (https://www.ncbi.nlm.hiv.gov/geo/) under accession numbers GSE280410 and GSE280411. Protein–protein interactions were retrieved from the STRING v.11 database (https://string-db.org/).

## Code availability

All original code is available from GitHub at https://github.com/AntoineFages/scRNA_paper.
