# Supplementary material for: Adaptive cellular evolution in the intestine of hyperdiverse cichlid fishes
Source: Nature. 2026 May 13;655(8122):401–8. doi: 10.1038/s41586-026-10494-8 (PMC13661267; doi:10.1038/s41586-026-10494-8)
Supplement: Supplementary file 1 [file 41586_2026_10494_MOESM1_ESM.pdf]

nature portfolio

Corresponding author(s): Antoine Fages, Walter Salzburger, Patrick Tschopp

Last updated by author(s): Apr 10, 2025

Reporting Summary

Nature Portfolio wishes to improve the reproducibility of the work that we publish. This form provides structure for consistency and transparency in reporting. For further information on Nature Portfolio policies, see our Editorial Policies and the Editorial Policy Checklist.

Statistics

For all statistical analyses, confirm that the following items are present in the figure legend, table legend, main text, or Methods section.

n/a	Confirmed	
☐	☒	The exact sample size (*n*) for each experimental group/condition, given as a discrete number and unit of measurement
☐	☒	A statement on whether measurements were taken from distinct samples or whether the same sample was measured repeatedly
☐	☒	The statistical test(s) used AND whether they are one- or two-sided<br>*Only common tests should be described solely by name; describe more complex techniques in the Methods section.*
☒	☐	A description of all covariates tested
☐	☒	A description of any assumptions or corrections, such as tests of normality and adjustment for multiple comparisons
☐	☒	A full description of the statistical parameters including central tendency (e.g. means) or other basic estimates (e.g. regression coefficient) AND variation (e.g. standard deviation) or associated estimates of uncertainty (e.g. confidence intervals)
☐	☒	For null hypothesis testing, the test statistic (e.g. *F*, *t*, *r*) with confidence intervals, effect sizes, degrees of freedom and *P* value noted<br>*Give P values as exact values whenever suitable.*
☐	☒	For Bayesian analysis, information on the choice of priors and Markov chain Monte Carlo settings
☒	☐	For hierarchical and complex designs, identification of the appropriate level for tests and full reporting of outcomes
☐	☒	Estimates of effect sizes (e.g. Cohen's *d*, Pearson's *r*), indicating how they were calculated

Our web collection on statistics for biologists contains articles on many of the points above.

Software and code

Policy information about availability of computer code

Data collection	No software was used for data collection.
Data analysis	Cell Ranger v5.0 (10X Genomics), Trimmomatic (v0.39), STAR (v2.7.3), HTSeq (v2.0), REVIGO web server (v1.8.1), EVEE-tools, STRING v11 database, Python3 (v3.12), oggmap Python package, BayesTraits (v3.0.2),R v4.1.2, R packages: SoupX (v1.6.2), ddqcR (v0.1), Seurat (v4.1.0), SciBet (v1.0), MetaNeighbor (v1.14), BayesPrism (v2.0), limma (v3.50.3), Go.db (v3.14), DESeq2 (v1.14), Augur (v1.0.3), caper (v1.0.3), speckle (v0.0.3), mvMORPH (v1.1.8), geiger (v2.0.7), coda (v.0.19.3), phytools (v2.1.1), ape (v5.7.1), ggplot2 (v3.5.1), scWGCNA, pheatmap (v1.0.12), dplyr (v1.1.4), reshape2 (v1.4.4), viridis (v0.6.5).<br>All original code is available on GitHub (https://github.com/AntoineFages/scRNA_paper).

For manuscripts utilizing custom algorithms or software that are central to the research but not yet described in published literature, software must be made available to editors and reviewers. We strongly encourage code deposition in a community repository (e.g. GitHub). See the Nature Portfolio guidelines for submitting code & software for further information.

Data

Policy information about availability of data

All manuscripts must include a data availability statement. This statement should provide the following information, where applicable:

- Accession codes, unique identifiers, or web links for publicly available datasets
- A description of any restrictions on data availability
- For clinical datasets or third party data, please ensure that the statement adheres to our policy

All single-cell and bulk RNA-seq raw and processed data generated in this study have been deposited at GEO (https://www.ncbi.nlm.hiv.gov/geo/) under accession numbers GSE280410 and GSE280411. The Nile tilapia reference genome used is available under RefSeq accession GCF_001858045.2.

Research involving human participants, their data, or biological material

Policy information about studies with human participants or human data. See also policy information about sex, gender (identity/presentation), and sexual orientation and race, ethnicity and racism.

Reporting on sex and gender	*Use the terms sex (biological attribute) and gender (shaped by social and cultural circumstances) carefully in order to avoid confusing both terms. Indicate if findings apply to only one sex or gender; describe whether sex and gender were considered in study design; whether sex and/or gender was determined based on self-reporting or assigned and methods used. Provide in the source data disaggregated sex and gender data, where this information has been collected, and if consent has been obtained for sharing of individual-level data; provide overall numbers in this Reporting Summary. Please state if this information has not been collected. Report sex- and gender-based analyses where performed, justify reasons for lack of sex- and gender-based analysis.*
Reporting on race, ethnicity, or other socially relevant groupings	*Please specify the socially constructed or socially relevant categorization variable(s) used in your manuscript and explain why they were used. Please note that such variables should not be used as proxies for other socially constructed/relevant variables (for example, race or ethnicity should not be used as a proxy for socioeconomic status). Provide clear definitions of the relevant terms used, how they were provided (by the participants/respondents, the researchers, or third parties), and the method(s) used to classify people into the different categories (e.g. self-report, census or administrative data, social media data, etc.) Please provide details about how you controlled for confounding variables in your analyses.*
Population characteristics	*Describe the covariate-relevant population characteristics of the human research participants (e.g. age, genotypic information, past and current diagnosis and treatment categories). If you filled out the behavioural & social sciences study design questions and have nothing to add here, write "See above."*
Recruitment	*Describe how participants were recruited. Outline any potential self-selection bias or other biases that may be present and how these are likely to impact results.*
Ethics oversight	*Identify the organization(s) that approved the study protocol.*

Note that full information on the approval of the study protocol must also be provided in the manuscript.

Field-specific reporting

Please select the one below that is the best fit for your research. If you are not sure, read the appropriate sections before making your selection.

☐ Life sciences ☐ Behavioural & social sciences ☒ Ecological, evolutionary & environmental sciences

For a reference copy of the document with all sections, see nature.com/documents/nr-reporting-summary-flat.pdf

Ecological, evolutionary & environmental sciences study design

All studies must disclose on these points even when the disclosure is negative.

Study description	We generated scRNAseq and bulkRNA data from the intestines of 25 African cichlid species, and leveraged that data to test for correlations between environment and phenotype at the cellular level. Specifically, we characterized the intestinal cell populations in these cichlid species and uncovered correlations between epithelial cell population relative abundances, cell-population-specific transcriptomic profiles, and the ecological/dietary niches that these species occupy.
Research sample	Our sample consists of specimens from 24 cichlid species from Lake Tanganyika and the Nile tilapia, used here as an evolutionary outgroup. All specimens were adults or subadults, and no manipulations were performed.
Sampling strategy	No statistical methods were performed to predetermine sample size, no sample-size calculations were performed. Sampling was designed to cover all phylogenetic sub-clades of cichlid fishes from Lake Tanganyika (so-called 'tribes') as well as all major feeding specializations.
Data collection	Individual fish dissections were performed by Navaneeth Menon, tissue dissociation into single-cell suspensions were performed by

Data collection	Antoine Fages and Maëva Luxey, single-cell capture, cDNA generation and genomic libraries were prepared by Antoine Fages, bulk RNA extractions were performed by Antoine Fages and Charlotte Huyghe.<br>Sequencing was performed on a Novaseq6000 instrument at the Genomics Facility Basel of the ETH Zurich Department of Biosystems Science and Engineering (D-BSSE).
Timing and spatial scale	Sampling and data collection were regularly performed at the Zoological Institute, University of Basel, between November 2020 and July 2025.
Data exclusions	Some samples were excluded from some cross-cell type and cross-species bioinformatic analyses due to low number of cells and/or sequencing reads in some cell populations.
Reproducibility	All experiments were performed following well-detailed protocols to ensure reproducibility of experimental findings.<br>All codes necessary to replicate the findings have been made publicly available on Github (https://github.com/AntoineFages/scRNA_paper).
Randomization	Randomization was not relevant to this study as no experimental groups were used.
Blinding	Blinding was not relevant for sampling and sequencing as an investigator bias can be ruled out, and no blinding was applied for data analyses as species and ecological information were relevant.

Did the study involve field work? ☐ Yes ☒ No

Reporting for specific materials, systems and methods

We require information from authors about some types of materials, experimental systems and methods used in many studies. Here, indicate whether each material, system or method listed is relevant to your study. If you are not sure if a list item applies to your research, read the appropriate section before selecting a response.

Materials & experimental systems

n/a	Involved in the study
☒	☐ Antibodies
☒	☐ Eukaryotic cell lines
☒	☐ Palaeontology and archaeology
☐	☒ Animals and other organisms
☒	☐ Clinical data
☒	☐ Dual use research of concern
☒	☐ Plants

Methods

n/a	Involved in the study
☒	☐ ChIP-seq
☒	☐ Flow cytometry
☒	☐ MRI-based neuroimaging

Animals and other research organisms

Policy information about studies involving animals; ARRIVE guidelines recommended for reporting animal research, and Sex and Gender in Research

Laboratory animals	The study involved sub-adult and adult cichlid fishes from the following species: Oreochromis niloticus, Boulengerochromis microlepis, Trematocara zebra, Bathybates ferox, Neolamprologus brevis, Lepidiolamprologus elongatus, Lepidiolamprologus attenuatus, Neolamprologus brichardi, Julidochromis ornatus, Telmatochromis vittatus, Cyphotilapia frontosa, Triglachromis otostigma, Callochromis pleurospilus, Xenotilapia spiloptera, Cyprichromis coloratus, Benthochromis horii, Haplotaxodon microlepis, Perissodus microlepis, Eretmodus cyanostictus, Astatotilapia burtoni, Tropheus moorii, Petrochromis orthognatus, Simochromis diagramma, Shuja horei and Limnotilapia dardennii.
Wild animals	The study did not involve wild animals.
Reporting on sex	Sex identification was not always possible and hence sex-based analyses were not performed.
Field-collected samples	The study did not involved field-collected samples.
Ethics oversight	All experiments were performed under animal experimentation permit 1010H and permit 2317 from the Cantonal Veterinary Office Basel Stadt.

Note that full information on the approval of the study protocol must also be provided in the manuscript.

Plants

Seed stocks	*Report on the source of all seed stocks or other plant material used. If applicable, state the seed stock centre and catalogue number. If plant specimens were collected from the field, describe the collection location, date and sampling procedures.*
Novel plant genotypes	*Describe the methods by which all novel plant genotypes were produced. This includes those generated by transgenic approaches, gene editing, chemical/radiation-based mutagenesis and hybridization. For transgenic lines, describe the transformation method, the number of independent lines analyzed and the generation upon which experiments were performed. For gene-edited lines, describe the editor used, the endogenous sequence targeted for editing, the targeting guide RNA sequence (if applicable) and how the editor was applied.*
Authentication	*Describe any authentication procedures for each seed stock used or novel genotype generated. Describe any experiments used to assess the effect of a mutation and, where applicable, how potential secondary effects (e.g. second site T-DNA insertions, mosiacism, off-target gene editing) were examined.*
